# Biomimetic materials: a promising strategy for periodontal tissue engineering and regeneration

**DOI:** 10.3389/fbioe.2025.1639170

**Published:** 2025-11-25

**Authors:** Qinghua Zhang, Ce Gou, Zhijun Zhang

**Affiliations:** 1 School of Clinical Medicine, Chengdu Medical College, Chengdu, China; 2 Department of Stomatology, The First Affiliated Hospital of Chengdu Medical College, Chengdu, China

**Keywords:** periodontal tissue regeneration, guided tissue regeneration, biomimetic materials, tissue engineering, extracellular matrix

## Abstract

Periodontitis is a chronic inflammatory disorder that causes irreversible damage to periodontal tissues, such as the alveolar bone, cementum, and periodontal ligament. Clinical treatments for periodontitis include non-surgical and surgical approaches, complemented by the utilization of various biomimetic materials to repair damaged tissues, a process known as periodontal tissue regeneration. Biomimetic materials have demonstrated extensive application prospects in periodontal tissue regeneration owing to their outstanding biocompatibility, controllable mechanical properties, and biomimetic microstructures. This review begins by delineating the cells used in periodontal tissue regeneration and summarizes the applications and research hot spots of commonly employed biomaterials in periodontal tissues. This further highlights the advantages of using biomimetic materials as bone substitutes, barrier membranes, and complex scaffold-manufacturing techniques. In addition, an in-depth discussion is conducted on their biological safety and physical properties. Moreover, this article summarizes the research advancements of extracellular matrix (ECM) materials in periodontal tissues, which hold great promise as highly prospective biomaterials for periodontal tissue regeneration. Finally, this article provides a comprehensive summary of the current limitations and challenges associated with the application of biomaterials in periodontal tissue regeneration. Although biomimetic materials still encounter challenges regarding stability and long-term efficacy during clinical translation, this review offers insights into the basic research and clinical treatment of periodontal regeneration.

## 1 Introduction

**FIGURE 1:**
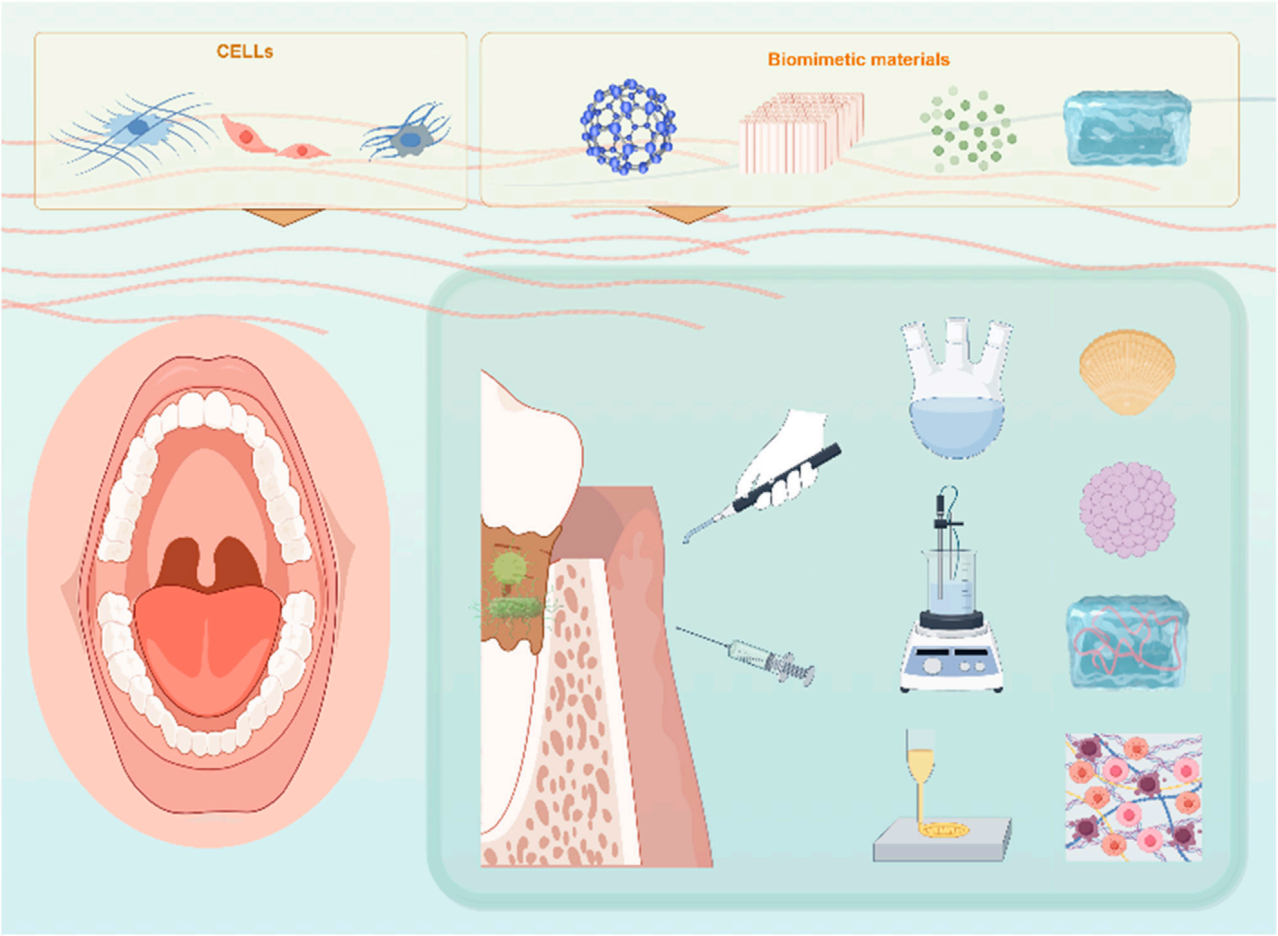
Biomimetic materials for periodontal tissue regeneration.

Periodontitis is a highly prevalent chronic inflammatory disorder that mainly affects the alveolar bone, cementum, and periodontal ligament, which are crucial tissues that support the tooth (Peng et al., 2024). Globally, approximately 743 million individuals are affected, with a higher prevalence in low-income groups and older populations (Eke et al., 2020; Papapanou and Susin, 2017). Approximately 42% of adults aged 30 years or older suffer from periodontal disease, and nearly 19% of the global adult population has severe periodontitis (Jain et al., 2024). Chronic periodontitis, characterized by excessive inflammatory responses, can lead to the progressive destruction of tooth-supporting structures, including the gums, periodontal ligament, and alveolar bone, ultimately resulting in tooth loss (Hajishengallis and Chavakis, 2021) (Figure 1). Conventional periodontal treatments, such as scaling, root planing, and open-flap debridement, aim to halt the progression of periodontitis; impaired periodontal tissues and alveolar bone remain largely irrecoverable following these interventions (Liang Y. et al., 2020; Yamada et al., 2022).

**FIGURE 2:**
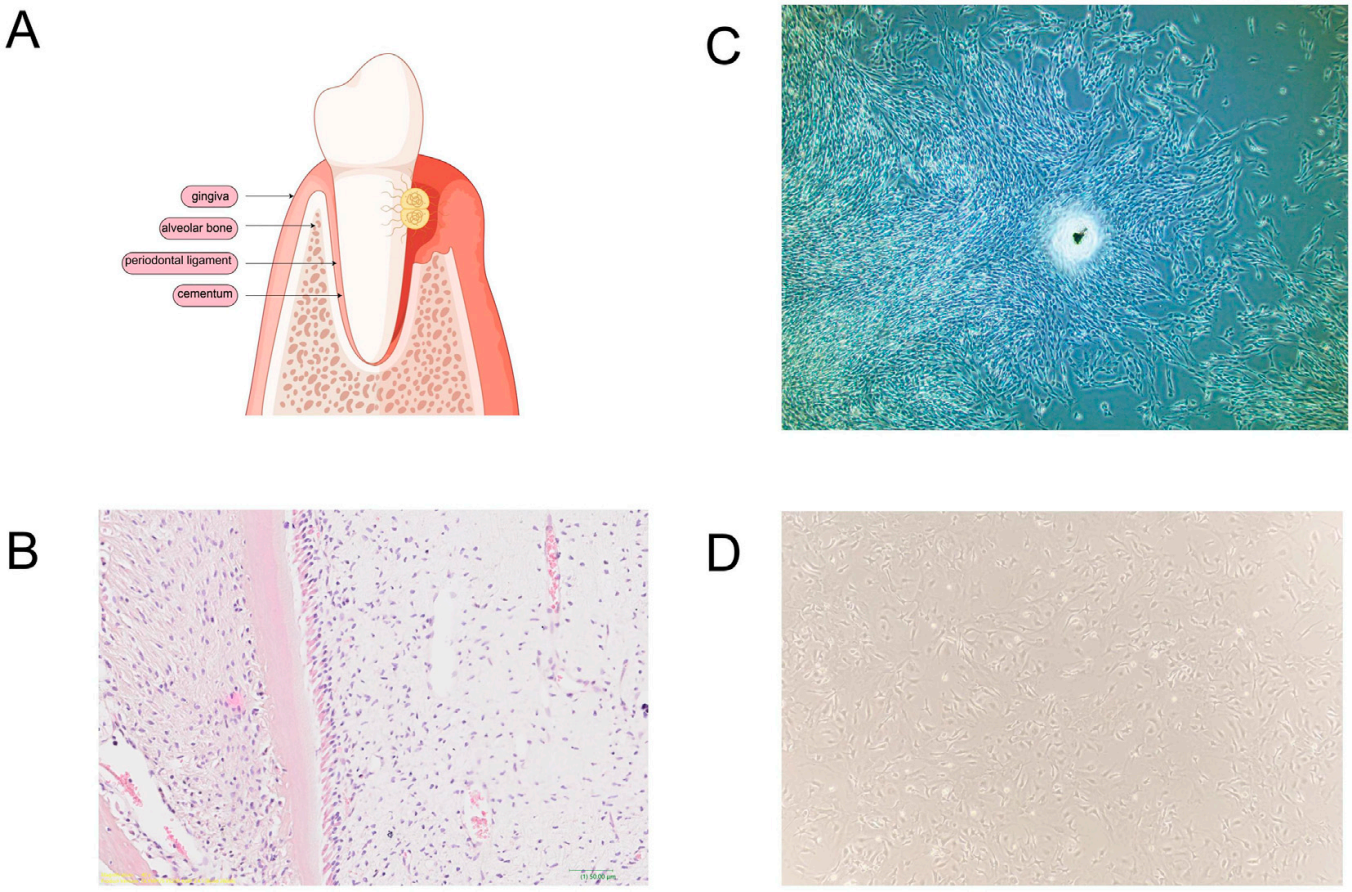
Periodontal tissues and cells suitable for periodontal tissue regeneration. **(A)** Tissue destruction of periodontal tissue and periodontitis. **(B)** Periodontal tissues of SD rats were stained with hematoxylin and eosin (H&E) for histological examination. **(C)** Dental follicle stem cells from beagle dogs. **(D)** Bone marrow-derived mesenchymal stem cells from C57 mice.

Periodontal tissues have diverse cell compositions and individualized tissue structures (Figures 2A,B). The key challenge in periodontal treatment is the restoration of the physiological structure and function of periodontal tissues. Techniques, such as guided tissue regeneration (GTR) and guided bone regeneration (GBR), show promise in repairing periodontal tissues and addressing alveolar bone defects (Abdo et al., 2023; Bottino et al., 2011; Ho et al., 2022). Current clinical approaches include GTR, bone grafting, and application of growth factors. However, these methods often suffer from limitations, such as poor predictability and restricted indications, resulting in unsatisfactory outcomes (Wu et al., 2025). Biomimetic materials can provide cells with a wide range of biochemical and biophysical signals that simulate the extracellular matrix (ECM) of the body. Moreover, these materials possess mechanical adaptability, microstructure connectivity, and intrinsic biological activity, making them an ideal choice for designing living implants for specific applications, covering the fields of tissue engineering and regenerative medicine (Liu et al., 2023). Scaffolds fabricated from biomimetic materials play a critical role in guiding cell proliferation and the formation of new tissue. Single-layer scaffolds, which consist of a uniform material structure, are inadequate for mimicking the complex biological environment of native tissues. In contrast, multi-layer scaffolds constructed from biomimetic materials can more effectively replicate the morphological architecture of tissues and organs, offering distinct advantages in the repair and regeneration of bone cartilage, skin, blood vessels, and various other tissue types (Bertsch et al., 2023). Due to the superior performance of biomimetic materials, a wide range of such materials has been approved for clinical use by the Food and Drug Administration (FDA) in tissue engineering, regeneration, and other biomedical applications (Table 1).

**TABLE 1:** Biomimetic materials that have been approved by the FDA.

Biomimetic material	Device	Reference
Hydroxyapatite (HA)	Biodegradable poly (octamethylene citrate) (POC)/hydroxyapatite-based orthopedic fixation devices	Xu et al. (2024)
Calcium phosphate (CaP) bioceramics	CaP coatings on orthopedic and dental endosseous implants	Eliaz and Metoki (2017)
Tricalcium phosphate (TCP)	mPCL–TCP scaffold loaded with autologous bone graft for bone regeneration	Laubach et al. (2022)
Ultrahigh molecular weight polyethylene (UHMWPE)	Total ankle and knee replacements	Jiang et al. (2023)
Polylactic acid (PLA)	Drug delivery; bone implants	Li et al. (2020)
Poly (glycolic acid) (PGA)	Bone regeneration	Katebifar et al. (2023)
Poly (lactic-co-glycolic acid) (PLGA)	PLGA-based nanoparticles used in drug delivery systems for parenteral administration	Danhier et al. (2012)
Hyaluronic acid	Orthopedic joint injection solution; soft tissue filler	Hemmati-Sadeghi et al. (2018), Ortiz et al. (2020)

With the application of tissue engineering technology in organ regeneration, biomimetic materials have been widely used for repairing tissue defects. Research into biomimetic materials holds the potential for developing new diagnostic and therapeutic strategies, enhancing the regeneration of damaged periodontal tissues, and providing avenues for restoring periodontal structure and function. This study reviewed eligible publications, identified based on inclusion/exclusion criteria, over a range of 29 years (from 1997 to 2025). This manuscript enumerates the cells utilized in periodontal regeneration research and presents a comprehensive review of the applications of biomaterials in periodontal regeneration. This study provides a theoretical foundation for basic research on periodontal tissue regeneration, thereby facilitating its clinical applications.

## 2 Stem cells used in periodontal tissue regeneration

Tissue engineering involves three key components: cells, scaffolds, and bioactive factors. Cells that secrete bioactive factors play critical roles in tissue repair and regeneration. Various dental-derived stem cells have been investigated for their potential in periodontal tissue regeneration, along with cells from other tissue sources, which are also of great significance in this field. Numerous types of stem cells have been investigated in animal models to assess their potential for periodontal tissue regeneration. This section focuses on the role of these cells in periodontal tissue regeneration (Table 2).

**TABLE 2:** Stem cells used in periodontal tissue regeneration.

Stem cells	Reference	*In vivo*/*in vitro*	Method	Result
PDLSCs	Lan et al. (2023)	*In vitro*	Constructing curcumin-primed PDLSC-derived extracellular vesicles (Cur-PDLSC-EVs) from cell culture supernatants of curcumin-pretreated PDLSCs	Promotes osteogenesis
	Li et al. (2024)	*In vitro*	OCCM-30 cementoblasts were cultured with various human periodontal ligament stem cell-derived exosome concentrations	Promotes the repair process in orthodontically induced inflammatory root resorption
	Zhao et al. (2022)	*In vivo* and *in vitro*	Investigating the effects of P-EVs on the proliferation and migration capabilities of bone marrow mesenchymal stem cells (BMMSCs) and exploring the potential mechanism of action	Accelerates bone tissue repair
	Lu et al. (2023)	*In vivo* and *in vitro*	Investigating the effects of these two types of PDLSCs–Exo on alveolar bone loss *in vivo* in mice with experimental periodontitis	Attenuates alveolar bone loss
DFSCs	Wei et al. (2023)	*In vivo* and *in vitro*	Regulating the periodontal immune microenvironment and providing a promising molecular agent	Promotes periodontal regeneration
	Shi et al. (2020)	*In vivo* and *in vitro*	An L-D-sEV-loaded hydrogel applied in the treatment of periodontitis was beneficial to repair lost alveolar bone in the early stage of treatment and maintain the level of alveolar bone in the late stage of treatment in experimental periodontitis rats	Promotes periodontal regeneration
	Ma et al. (2022)	*In vivo* and *in vitro*	The effect of DFSCs–sEVs on the biological behavior of PDLSCs was examined using the EdU assay, the CCK-8 assay, cell cycle analysis, wound healing, alizarin red staining, qRT-PCR, and Western blot analysis	Promotes periodontal tissue regeneration
	Liu L. et al. (2024)	*In vivo* and *in vitro*	The biological characteristics and functions of E-DFSCs were verified by proliferation, differentiation, and co-culture experiments	Promotes the healing and regeneration of periodontal tissue
SHED	Wang M. et al. (2020)	*In vitro*	SHED-Exos promoted PDLSC osteogenic differentiation with deep alizarin red staining, high alkaline phosphatase (ALP) activity, and upregulated osteogenic gene expression (RUNX2, OPN, and OCN)	Enhances the osteogenic ability of PDLSCs
	Wei et al. (2020)	*In vivo*	SHED-Exos specifically promoted BMSC osteogenesis and inhibited adipogenesis compared with SHED-derived conditioned medium	Promotes bone formation
	Wu et al. (2019)	*In vivo* and *in vitro*	The impacts of SHED-derived exosomes on the angiogenic ability of human umbilical vein endothelial cells (HUVECs) and the osteogenic capability of rat bone marrow mesenchymal stem cells (BMSCs) were evaluated	Contributes to periodontal bone regeneration
BMSCs	Liu L. et al. (2021)	*In vivo* and *in vitro*	The human periodontal ligament cells (hPDLCs) were co-cultured with BMSC-sEV *in vitro* to detect the effects of BMSC-sEV on hPDLC migration, proliferation, and differentiation	Promotes periodontal tissue regeneration
	Li et al. (2022)	*In vivo* and *in vitro*	ApoEVs promoted new bone formation by increasing intracellular reactive oxygen species to activate JNK signaling	Promotes bone regeneration in the calvarial defect
ADSCs	Yang et al. (2022)	*In vivo* and *in vitro*	hADSCs delivered CGRP to hPDLSCs through EVs, thereby promoting the osteogenic differentiation potential of hPDLSCs	Induces bone defect repair

### 2.1 Periodontal ligament stem cells

A distinct population of multipotent postnatal stem cells resides in the periodontal ligament, where they contribute to tissue homeostasis and regeneration. Periodontal ligament stem cells (PDLSCs) were initially isolated using a range of scientifically validated techniques and comprehensively characterized by Seo et al. (2004) and Trubiani et al. (2005). PDLSCs are somatic stem cells with the potential to differentiate into multiple cell types and undergo robust clonal self-renewal (Coura et al., 2008; Dapeng et al., 2014; Li et al., 2014).

### 2.2 Dental follicle stem cells

Dental follicle stem cells (DFSCs), originating from the neural crest and located in the dental follicle tissue of the tooth germ, serve as direct precursors to periodontal tissues (Figure 2C) (Zhang et al., 2019). These multipotent stem cells differentiate into osteoblasts. Initially identified by Morsczeck et al. (2005), DFSCs are precursor cells for periodontal tissues. They can develop into the periodontal ligament, cementum, and alveolar bone under specific conditions during the late stage of tooth development (Liu L. et al., 2024; Shi et al., 2020).

### 2.3 Stem cells from human exfoliated deciduous teeth

Stem cells from human exfoliated deciduous teeth (SHED) are a highly proliferative and clonogenic cell population capable of differentiating into various cell types, including neural cells, adipocytes, and odontoblasts (Yan et al., 2021). Miura first discovered that exfoliated human deciduous teeth contain these multipotent stem cells (Miura et al., 2003). SHED-derived exosomes combined with β-tricalcium phosphate (TCP) promote alveolar bone regeneration by enhancing angiogenesis and osteogenesis (Wu et al., 2019). Owing to their multipotent nature, similar to that of mesenchymal stem cells (MSCs), SHED hold significant promise in regenerative medicine (Mohd Nor et al., 2023).

### 2.4 Bone mesenchymal stem cells

Multipotent stem cells reside in both the adipose tissue and bone marrow, with bone mesenchymal stem cells (BMSCs) being predominant (Figure 2D) (Luo P. et al., 2023). These adult stem cells exhibit multi-differentiation, self-renewal, migration, and colonization abilities (Hade et al., 2021). As initiator cells, BMSCs promote periodontal tissue regeneration and possess immunoregulatory properties crucial for the periodontal inflammatory response (Song et al., 2007; Zhang et al., 2018). In dental applications, BMSCs have demonstrated significant efficacy in periodontal regeneration (Kawaguchi et al., 2004).

### 2.5 Adipose-derived stem cells

Adipose-derived stem cells (ADSCs) are a population of multipotent stromal cells extracted from the SVF of subcutaneous adipose tissue. These cells exhibit a self-renewal capacity and the potential to differentiate into diverse cell lineages, making them attractive candidates for tissue repair and regeneration (Gou et al., 2024; Sándor et al., 2014; Si et al., 2019). ADSCs exert their therapeutic effects by releasing paracrine factors and extracellular vesicles (EVs) that modulate the local microenvironment and stimulate endogenous regenerative processes (Zhang et al., 2020). Numerous studies have reported the successful application of ADSCs in the regeneration of the alveolar bone, a critical component of the oral and maxillofacial complex (Lemaitre et al., 2017; Yang et al., 2022; Zhang et al., 2025).

## 3 Biomimetic materials used in periodontal tissue regeneration

**FIGURE 3:**
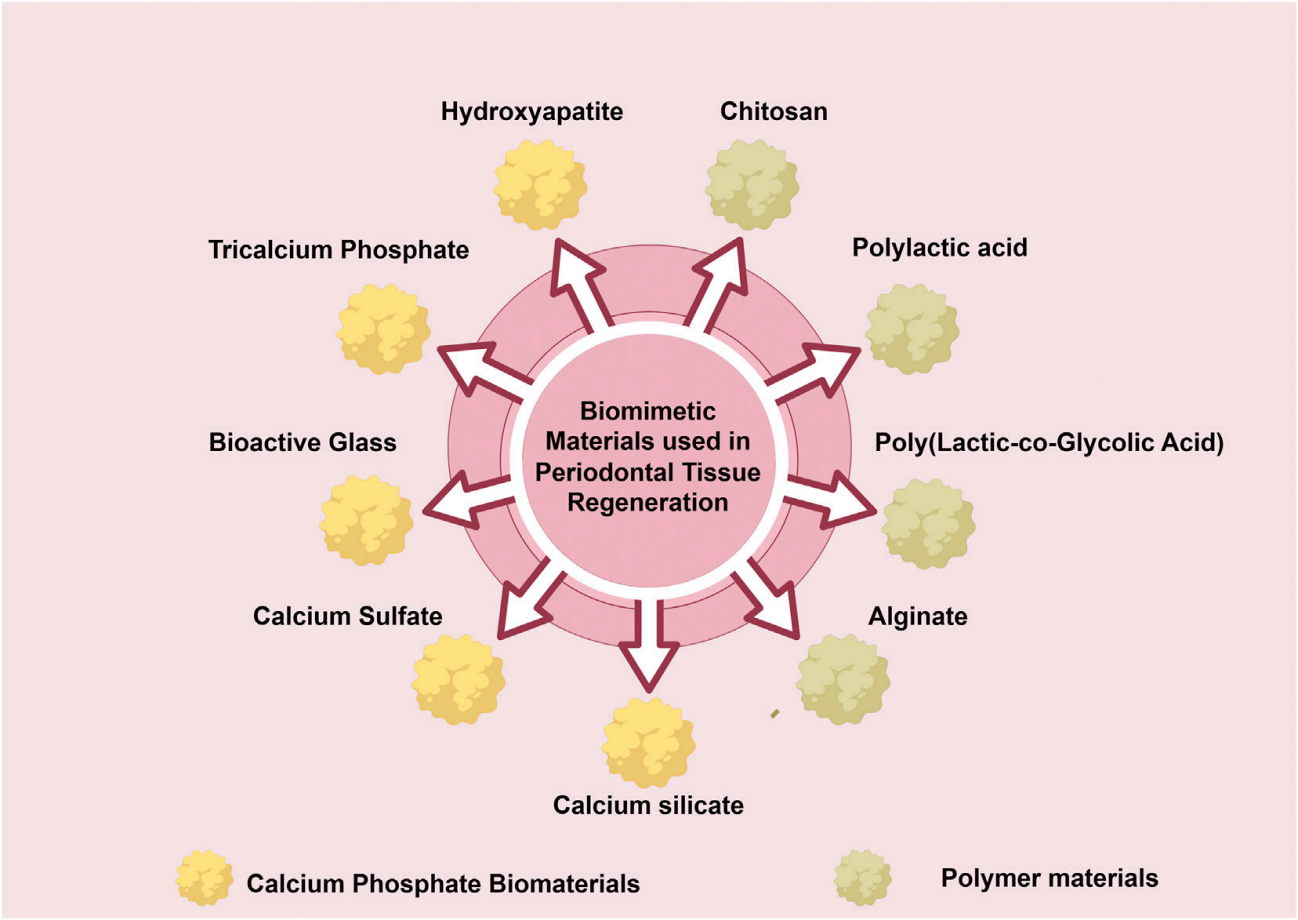
Biomimetic materials for periodontal tissue regeneration.

Bioactive nanomaterials have significantly transformed the field of bone tissue regeneration, effectively addressing the limitations of autogenous and allogeneic grafts (Figure 3). Engineered scaffolds and nanocomposites with high biocompatibility, such as calcium phosphate (CaP) composites, thermosensitive hydrogels, and hydroxyapatite, play crucial roles in facilitating bone repair (Hao et al., 2024). The micro and nanoscale characteristics of natural bone, including porosity, surface topography, and fiber alignment, necessitate reevaluation in order to design scaffolds that effectively stimulate tissue growth (Scott et al., 2013). An interconnected 3D pore architecture is essential for bone tissue scaffolds as it facilitates adequate cell accommodation, the migration of osteoprogenitor cells and immune cells, vascularization, and innervation. The optimal size range for micropores to promote cellular infiltration and attachment within bone tissues is generally considered to be between 50 and 150 µm (Yan et al., 2024). In contrast, macropores ranging from 100 to 600 µm provide enhanced integration with host bone tissue while supporting vascularization and new bone formation (Zhu et al., 2020).

### 3.1 CaP biomaterials

CaP-based materials have garnered considerable interest in implant dentistry because of their superior osteoconductivity and bone-regenerative capabilities (Huang et al., 2017; Yang et al., 2011). Their chemical and structural resemblance to natural bone makes them ideal for repairing bone defects (Mohd Pu’ad et al., 2019). Following alkali treatment, the scaffolds were immersed in a pre-established solution to allow coating formation. The presence of nanostructured F/CaP coating led to a marked upregulation of osteogenic genes and attenuated bacterial growth. While human periodontal ligament stem cells (hPDLSCs) on the scaffolds with 500 μm strand spacing showed high expression of osteogenic markers (runt-related transcription factor 2, Runx2) over 21 days, cells on randomly oriented fibrous scaffolds showed upregulation of M1 markers (Daghrery et al., 2023). *In vivo* findings confirmed that F/CaP-coated scaffolds were biocompatible and led to periodontal regeneration when implanted in a rat mandibular periodontal fenestration defect model (Daghrery et al., 2021). The biodegradation of calcium phosphate is influenced by various factors, including porosity, the extent of bone contact, specific surface area, bone type, and animal species (Lu et al., 2002). This section reviews the recent developments, characterization, and biological performance of different CaP-based materials for periodontal tissue regeneration (Table 3).

**TABLE 3:** Calcium phosphate biomimetic materials used in periodontal tissue regeneration.

Material	Reference	*In vivo*/*in vitro*	Biocompatibility	Method	Result
HA	Mao et al. (2015)	*In vitro*	Promotes cell adhesion, proliferation, and alkaline phosphatase (ALP) activity	HA bioceramics with a micro-nano-hybrid surface (mnHA [the hybrid of nanorods and microrods]) were fabricated via hydrothermal reaction of the α-tricalcium phosphate granules as precursors in an aqueous solution	Promotes periodontal tissue regeneration
	Chang et al. (2021)	*In vitro*	Assists angiogenesis, the formation of calcium-containing matrix vesicles, and mineral crystallization, providing sufficient stiffness that directs cells to differentiate into the osteogenic lineage	Scaffolds composed of 90% hydroxyapatite and 10% poly (lactic-co-glycolic acid) were printed using a microextrusion process to fit 4-mm-diameter and 0.5-mm-thick through-and-through osseous defects on the mandibular ramus of rats, with unfilled defects serving as controls	Accelerates bone formation
	Conner et al. (2003)	*In vitro*	—	Mandibular premolars and first molars were extracted in 12 dogs, and healing was allowed for 6 months. Ten-mm-long titanium screw-type implants with titanium plasma-sprayed (TPS), hydroxyapatite-coated (HA), or acid-etched (AE) surfaces were placed	Promotes bone regeneration
TCP	Fakheran et al. (2019)	*In vivo*	Allows cellular adhesion, growth, and proliferation on its surface	Retro MTA covered with a collagen membrane, Retro MTA + β-TCP covered with a membrane, and the defect covered with a membrane without any bone augmentation	Promotes periodontal tissue regeneration
	Nagahara et al. (2015)	*In vivo*	Promotes differentiation of the cells into osteoblasts to ameliorate delayed bone regeneration	MSC/Col/TCP or MSC/Col was transplanted into experimental periodontal Class III furcation defects that had been exposed to inflammation in beagle dogs	Promotes the augmentation of the alveolar bone
	Yoshino et al. (2023)	*In vivo*	Promotes proliferation of several types of cells, such as mesenchymal stromal cells and synovial mesenchymal cells	b-TCP dispersion (1 wt%; 500 mL) was added to 100 mg of RCP granules to form b-TCP/RCP. A three-walled intrabony defect (5 mm × 3 mm × 4 mm) was created on the mesial side of the mandibular first molar and filled with b-TCP/RCP	Promotes periodontal tissue regeneration
	Uchikawa et al. (2021)	*In vivo*	Promotes bone regeneration with mesenchymal stem cells (MSCs) by increasing alkaline phosphatase activity and upregulating the expression of the osteogenesis-related genes	The upper first or second molar of a 3-week-old C57BL/6J mice and a β-tricalcium phosphate (β-TCP) scaffolds were transplanted with BM-MNCs (the MNC group) or without BM-MNCs (the β-TCP group) into the thigh muscle of syngeneic mice	Accelerates bone formation and periodontal tissue regeneration
BG	Villaça et al. (2005)	*In vivo*	Promotes cellular proliferation, differentiation, apoptosis, and migration	The two-wall defects were made in the mesial area of the left and right second premolars of four monkeys, filled with gutta-percha, and after 15 days, they were debrided and either naturally filled with coagulum (control) or implanted with bioactive glass (test)	Promotes periodontal tissue regeneration
	Subbaiah and Thomas (2011)	*In vivo*	—	Use the guided tissue regeneration model in dogs	Promotes the formation of mineralized bone
CSi	Chen H. et al. (2016)	*In vivo*	Stimulates bone mesenchymal stem cell formation and differentiation	A radial bone defect model was constructed through an osteotomy using New Zealand rabbits	CS-PRP functions as an effective treatment for long bone defects by stimulating bone regeneration and enhancing new bone strength
	Li et al. (2023)	*In vivo* and *in vitro*	—	The immune response of CMC/OPC bone cement *in vitro* and *in vivo*	The CMC/OPC possesses biodegradable, anti-inflammatory, and osteogenic properties and can be employed as an attractive bone cement biomaterial for bone regeneration

#### 3.1.1 Hydroxyapatite

Hydroxyapatite (HA) has been a focal point of research since the 1980s because of its chemical resemblance to bone minerals, notable biological activity, and osteoconductivity (Dunaev et al., 2014; Lei et al., 2017). Stoichiometric HA features a dense crystalline network of PO_4_ tetrahedra, each centered around a P^5+^ ion coordinated by four oxygen atoms. These tetrahedra form interconnected columns, resulting in two distinct types of unconnected channels within the lattice. HA is commonly synthesized via methods such as solution–precipitation and sol–gel (Ben-Arfa et al., 2017; Mirzaee et al., 2016). These characteristics render HA a preferred bioceramic for periodontal bone tissue repair. Porous chitosan (CS) membranes were fabricated with or without HA using the simple freeze gelation (FG) technique with two different solvent systems: acetic acid (ACa) and ascorbic acid (ASa). Fourier-transform infrared (FT-IR) spectroscopy confirmed the presence of hydroxyapatite and its interactions with chitosan. All the membranes supported cell proliferation, and long-term matrix deposition was supported by the HA-incorporated membranes. These CS and HA composite membranes have potential for use in GTR for periodontal lesions (Qasim et al., 2015). Small intestinal submucosa (SIS) was coated with hydroxyapatite (SIS-HA) and gelatin methacrylate hydroxyapatite (SIS-Gel-HA) using biomineralization and chemical crosslinking. SIS was found to upregulate M2 marker expression, and both SIS-HA and SIS-Gel-HA enhanced the osteogenic differentiation of PDLSCs through the bone morphogenetic protein (BMP)-2/Smad signaling pathway, demonstrating superior *in vitro* osteogenic activity. *In vivo*, SIS-HA and SIS-Gel-HA yielded denser and more mature bone, with the highest BMP-2 and Smad expression. SIS-HA and SIS-Gel-HA demonstrated enhanced immunosteogenesis coupling, representing a promising periodontal tissue regeneration approach (Cheng et al., 2024). HA bioceramics with micro-nano-hybrid surfaces (mnHA [a hybrid of nanorods and microrods]) can promote cell adhesion, proliferation, alkaline phosphatase (ALP) activity, and expression of osteogenic/cementogenic-related markers, including Runx2, ALP, osteocalcin (OCN), cementum attachment protein (CAP), and cementum protein (CEMP). Various strategies have been employed to fabricate scaffolds with differing pore parameters (Carotenuto et al., 2022).

The TIPS technique, supported by the salt-leaching process, was employed to investigate the influence of HA content in PLLA/HA scaffolds on various properties, including pore structure, density, porosity, mechanical characteristics, and osteoblast proliferation (Zeinali et al., 2021). It was found that an increased HA content within the scaffold (up to 75 wt%) correlated with enhanced roughness of the pore wall surfaces, elevated porosity (96%–98%), and a higher proliferation rate of osteoblast cells. A more recent study conducted by the same research group demonstrated that apatite whiskers (HAP) completely covered the pore wall surfaces of PCL/HAP scaffolds, resulting in significantly increased surface roughness (Petersen et al., 2018). These scaffolds exhibited high porosity (approximately 90%) along with heterogeneous pores characterized by two distinct size ranges: larger pores with diameters of up to 600 µm and smaller pores located within micropore walls measuring up to 50 µm. Moreover, L-lysine modification of hydroxyapatite enhances the bioactivity of PCL/HAP scaffolds by promoting osteoblast proliferation and differentiation while simultaneously improving mechanical properties and increasing surface roughness of the wall pores. Compared to smooth surfaces, it is suggested that a rough topography may more effectively mimic the mineralized interface encountered by cells adhered to the native bone ECM. These results suggest that HA bioceramics containing mnHA are promising grafts for periodontal tissue regeneration (Mao et al., 2015).

#### 3.1.2 TCP

TCP, a well-studied calcium phosphate, exists in three polymorphs: α, β, and α′, each stable at different temperatures and notable for their bioactivity and degradability (Li et al., 2017). Calcium phosphates are favored as bone substitutes because of their biocompatibility and osteointegration (Stavropoulos et al., 2010). Although sintered hydroxyapatite is widely used in bone-tissue engineering, it is minimally resorbed. In contrast, β-TCP offers superior resorbability in both animal and human studies (Kon et al., 2000). Its three-dimensional structure enhances cell attachment, proliferation, and osteogenic differentiation (Nade et al., 1983; Zambonin et al., 2000). In a recent study, porous β-tricalcium phosphate (β-TCP) scaffolds were fabricated by integrating digital light processing (DLP) printing techniques with an *in situ* crystal growth process. These bioceramic scaffolds, featuring both macro- and micropores, were produced through DLP printing. Subsequently, an *in situ* crystal growth process was employed to generate micro-/nano-scale surface topographies. The resulting micro-/nanostructured scaffold enhanced the proliferation and differentiation of rat bone mesenchymal stem cells (rBMSCs), demonstrating significant capacity for skull bone regeneration in a rat model (Di Luca et al., 2016). Native bone tissue exhibits a structural gradient that can be observed radially in long bones and axially in flat bones due to variations in bone density and pore parameters from cancellous to cortical bone. Drawing inspiration from this natural architecture, Di Luca et al. developed three-dimensional polycaprolactone (PCL) scaffolds that feature an axial gradient in pore size and overall porosity using the “fused deposition modeling” (FDM) method. This scaffold notably enhanced osteogenic differentiation of human mesenchymal stem cells (hMSCs), particularly benefiting those residing within larger pores (Diloksumpan et al., 2020). Additionally, three-dimensional printing technology was utilized to create two types of 3D bioceramic scaffolds with distinct internal architectures—either exhibiting a designed porosity gradient or maintaining a uniform pore distribution. Nery and Lynch (1978) first reported the use of TCP-based bone grafts for periodontal defects in 1978. Owing to its high biodegradability, osteoconductivity, and osteoinductivity, β-TCP is a promising scaffold material for bone tissue engineering and is frequently used as an alternative to autologous bone grafts (Zhang J. et al., 2014). β-TCP microspheres with different diameters were fabricated via a solid-in-oil-in-water (S/O/W) emulsion method. BMSCs were co-cultured *in vitro* with β-TCP microspheres, and SEM and confocal microscope analyses were performed to discover that β-TCP microspheres efficiently promoted BMSC attachment and bone-related gene expression. The co-cultured BMSCs and microspheres were successfully implanted subcutaneously into nude mice for 8 weeks, and abundant new bone-like structures had formed between the β-TCP microspheres, implying that β-TCP microspheres can be used as a cell carrier and bone graft substitute material (Li et al., 2017).

The extensive application of β-TCP in bone tissue regeneration demonstrates its promising potential for periodontal tissue regeneration. The β-TCP scaffold seeded with bone marrow mononuclear cells (BM-MNCs) from C57BL/6J mice, when transplanted into the thigh muscle of syngeneic mice, demonstrated accelerated bone formation and enhanced periodontal tissue regeneration (Uchikawa et al., 2021). Owing to its excellent physical and chemical properties, β-TCP can be integrated with various biomimetic materials to enhance periodontal tissue regeneration. Different concentrations of β-TCP-loaded chitosan hydrogels (0%, 2%, 4%, or 6% β-TCP, 10% β-glycerol phosphate, and 1.5% chitosan) with cells can promote periodontal tissue regeneration (Tan et al., 2023). In the periodontal defect model of beagle dogs, the application of Retro mineral trioxide aggregate (MTA) or a combination of Retro MTA + β-TCP covered with a collagen membrane led to the regeneration of periodontal tissues (Fakheran et al., 2019).

#### 3.1.3 Bioactive glass

Bioactive glass (BG), a class of synthetic alloplastic materials with a silicate base, possesses the distinctive capability to bond with mineralized hard tissues, such as bone, in physiological environments. Developed by Hench et al. in 1969, BG marked a significant advancement in dental applications during the mid-1980s when Clark et al. successfully utilized it in a clinical trial for alveolar ridge preservation in edentulous patients (Välimäki and Aro, 2006). It was not until the 1990s, however, that BG was first used for the treatment of periodontal lesions (Zamet et al., 1997). Recent research has identified BG as an osteoinductive agent capable of inducing osteoprogenitor cell migration into graft structures and promoting cell differentiation by influencing the gene expression of undifferentiated cells (Kargozar et al., 2018; Kargozar et al., 2017). It demonstrated bioactivity along with a relatively high mechanical strength that exhibited only a gradual decline, even under load-bearing conditions within the body (Shue et al., 2012). Schepers and Ducheyne (1997) introduced the term “osseostimulation” to describe the bone-formation process facilitated by BG (Schepers and Ducheyne, 1997). The most extensively used BG over the past 50 years is 45S5 glass, which is characterized by a low SiO_2_ content and high Na_2_O and CaO contents, along with a high CaO/P_2_O_5_ ratio, enhancing its reactivity with biological fluids (Vitale-Brovarone et al., 2009). In dental applications, 45S5 glass particulate, commercially known as PerioGlas® (NovaBone Products LLC, Alachua, FL, United States), has been demonstrated to inhibit epithelial cell down-growth and promote alveolar bone regeneration (Mengel et al., 2003; Subbaiah and Thomas, 2011). A multi-functional and sustained-release drug delivery system (MB/BG@LG) was developed by encapsulating methylene blue (MB) and BG into a lipid gel (LG) precursor using Macrosol technology. In addition, *in vitro* and *in vivo* experiments showed that MB/BG@LG can effectively promote periodontal tissue regeneration by reducing the inflammatory response and promoting cell proliferation and osteogenic differentiation (Chen et al., 2023). The incorporation of nano-BG glass-ceramic particles into alginate composite scaffolds enhances the ALP activity of human periodontal ligament fibroblast cells cultured on these scaffolds. Mesoporous bioactive glass (MBG) is extensively utilized in bone tissue repair and drug delivery applications. However, the occurrence of burst release of drugs and poor compatibility with other materials has limited its broader application. Modifying MBG with a polymer brush presents an effective strategy to enhance its properties. In this study, an alginate (ALG)-modified MBG was synthesized, and the effects of ALG on the characteristics of MBG were systematically investigated. The results demonstrate that ALG significantly improves drug loading efficiency, prolongs drug release duration, and facilitates the orderly deposition of apatite on the surface of MBG (Yao et al., 2022). These results suggest that biocompatible composite scaffolds are promising for periodontal tissue regeneration (Srinivasan et al., 2012).

#### 3.1.4 Calcium sulfate

Calcium sulfate (CaS), commonly referred to as “plaster of Paris,” was first introduced as a synthetic bone graft material by Van Meekeren in 1892. This compound exists in three forms differentiated by the number of water molecules within its crystalline structure: anhydrates, dihydrates, and hemihydrates. The hemihydrate form, which dissolves rapidly, is predominantly utilized in medical-grade products and is available in two variants, α and β. Upon complete degradation in biological fluids, calcium sulfate leaves calcium phosphate deposits that promote bone growth (Shaffer and App, 1971; Zhao et al., 2021). Currently, calcium sulfate and its composites are primarily employed in dentistry and maxillofacial surgery as injectable bone fillers for sinus augmentation and alveolar bone regeneration in small periodontal defects (Thomas and Puleo, 2009).

#### 3.1.5 Calcium silicate

Because of their predominant biocompatibility, bioactivity, and sealing ability, calcium silicate (CSi)-based bioceramics have been widely used in endodontic treatment, including MTA, Biodentine, BioAggregate, and iRoot BP Plus (Parirokh et al., 2018). MTA is characterized by low or no toxicity and excellent biocompatibility. It stimulates repair by allowing cellular adhesion, growth, and proliferation on its surface. MTA possesses good antibacterial properties, but it does not exhibit significant cytotoxic effects on host cells. They possess a large specific surface area and a nanoporous or hollow structure, which contribute to their elevated drug-loading capacity. Additionally, these materials demonstrate pH-responsive drug release behavior and favorable drug release characteristics. Consequently, they hold significant promise for applications in drug delivery. The calcium silicate-based drug delivery systems are characterized by prolonged drug-release times, which can considerably extend the therapeutic effects of administered drugs. Another notable advantage of these systems is their pH-responsive nature regarding drug release; this feature renders them an ideal platform for targeted drug delivery (Zhu et al., 2017). Additionally, it induces cementogenesis while regenerating the periodontal ligament, leading to bone formation.

In recent years, several CSi-based cements have been proven to be potential bioactive materials for bone-substitute applications, and some of them exhibit excellent *in vitro* and *in vivo* osteogenesis and odontogenesis (Chen Y. W. et al., 2016). CSi is a common material with excellent mechanical strength (Zhang W. et al., 2021). In addition, CSi cement not only exhibits a good osteoconduction effect but also reduces inflammatory markers in primary human dental pulp stem cells (hDPSCs). CSi-based cements can also stimulate the osteogenic differentiation of various stem cells, such as bone marrow stromal cells, adipose-derived stem cells, human dental pulp cells, and periodontal ligament cells. CSi and β-TCP composite collagen membrane (Retro MTA + β-TCP) has been shown to effectively promote periodontal tissue regeneration in the periodontal defect model of beagle dogs, demonstrating significant potential for periodontal bone regeneration (Fakheran et al., 2019). A fast-setting and controllable degradation of magnesium–calcium silicate (Mg–CSi) cement should be developed via the sol–gel method, and a mechanism should be established to stimulate hPDLSCs using Mg ions. The proliferation, alkaline phosphatase, odontogenesis-related gene (*DSPP* and *DMP-1*), and angiogenesis-related protein (vWF and ang-1) secretion of hPDLSCs significantly increased when the Mg content of the specimen was increased. The results of this study suggest that Mg–CSi materials with this modified composition could stimulate hPDLSC behavior and serve as effective bioceramics for bone substitutes and hard tissue regeneration applications, as they stimulate odontogenesis/angiogenesis (Chen et al., 2015; Chen Y. W. et al., 2016).

### 3.2 Polymer materials

#### 3.2.1 Chitosan

CS, a linear polysaccharide derived from chitin, exhibits notable properties, such as biodegradability, biocompatibility, antimicrobial effects, low antigenicity, hygroscopicity, and moisturizing properties (Bee and Hamid, 2025). Typically sourced from crustacean exoskeletons, chitosan comprises randomly dispersed β-(1,4)-glucosamine and N-acetyl-D-glucosamine units. The deacetylation process of chitin, first identified by Rouget in 1859 and later termed “chitosan” by Hoppe-Seyler in 1894, has demonstrated significant biomedical applications (Duymaz et al., 2025). In recent years, CS has been advocated for GTR/GBR applications because of its exceptional properties, including biocompatibility, biodegradability, anti-inflammatory and antibacterial properties, hydrophilicity, mucoadhesive characteristics, membrane-forming ability, flexibility, and cost-effectiveness. CS has been demonstrated to facilitate cellular adaptation and proliferation of various essential cell types involved in regenerating periodontal tissue, such as osteoprogenitor cells, osteoblasts, periodontal ligament fibroblasts, and gingival fibroblasts. Seol et al. (2004) demonstrated that CS sponges enhance the proliferation and differentiation of osteoblasts. Similarly, Klokkevold et al. (1996) verified that CS promotes the differentiation of osteoprogenitor cells, specifically MSCs, and potentially aids osteogenesis and bone formation. CS nanogels showed no cytotoxicity toward hGF cells at concentrations of 10–80 μL/mL (M et al., 2023).

In a separate study, CS promoted hPDL cell proliferation at concentrations of 2,000, 1,000, 100, and 50 μg/mL compared to the control group. The morphology of hPDL cells cultured with CS indicated no apoptosis, highlighting its non-toxic properties. Additionally, CS supported initial osteoblast attachment and spreading over fibroblasts, which could be advantageous in GBR procedures for bone regeneration by promoting osteoblast attachment and proliferation. In patients with periodontitis, chitosan implantation has been shown to reduce gingival inflammation due to its antimicrobial characteristics (Xu C. et al., 2012). Yeo et al. (2005) illustrated that a nonwoven chitosan membrane effectively enhances bone and cementum regeneration in surgically induced one-wall intrabony defects in beagle dogs, underscoring its potential in GTR and GBR. Furthermore, a CS/MW/Mel scaffold, a blend of chitosan and mesoporous wollastonite loaded with melatonin, was investigated for biomedical applications (Wang et al., 2025). The mHA/CS scaffold demonstrated enhanced osteoblast differentiation at both the cellular and molecular levels, as evidenced by the increased expression of key osteogenic markers, such as Runx2, Type I collagen, and osteocalcin. CS possesses certain inherent disadvantages, including a high degradation rate and low mechanical strength. When hydroxyapatite is combined with chitosan to form a composite scaffold, the resulting material retains the advantageous properties of enhanced biocompatibility and improved mechanical strength (Rodríguez-Vázquez et al., 2015). Additionally, the scaffold exhibited proangiogenic properties, facilitating vascularization, which is essential for tissue regeneration. This study further revealed the inhibitory effects of the mHA/CS scaffold on the growth of periodontal pathogens. When loaded with 20 μg/mL of recombinant human amelogenin (rhAm), the composite scaffold significantly boosted ALP activity and upregulated the expression of Runx2, OPN, and DLX-5 genes and proteins *in vitro* (*p* < 0.05). The GdPO_4_/Fe_3_O_4_/chitosan hybrid scaffold facilitated tumor ablation by enabling rapid heating under near-infrared laser irradiation. The mesoporous structure exhibited a diameter of 7 nm, and its specific surface area measured 33.95 m^2^/g (Liao et al., 2020). Their large pores and directional structure promoted cell migration and attachment, thereby enhancing bone tissue regeneration via the BMP-2/Smad/runt-related transcription factor 2 pathway (Merkel et al., 2023; Zhao et al., 2020). Furthermore, the scaffold effectively stimulated the formation of cementum-like tissues *in vivo* (Liao et al., 2020). These findings suggest that the mHA/CS scaffold loaded with 20 μg/mL rhAm has the potential to inhibit the growth of periodontal pathogens while promoting the formation of bone and cementum-like tissues (Table 4).

**TABLE 4:** Polymer materials used in periodontal tissue regeneration.

Material	Reference	*In vivo*/*in vitro*	Biocompatibility	Method	Result
Chitosan	Yeo et al. (2005)	*In vivo*	Differentiation of undifferentiated mesenchymal cells into cementoblasts, and by promoting the differentiation of osteogenic cells	One-wall intrabony defects were surgically created bilaterally in the mandibular second and fourth premolars of six beagle dogs	Promotes periodontal regeneration
	Lee et al. (2016)	*In vivo*	Enhances the adhesion and proliferation of human gingival fibroblasts	The EGCG14-CS-Lovastatin1 membrane shows bone regeneration in one-wall defects of beagle dogs	Promotes periodontal regeneration
	Liao et al. (2020)	*In vivo* and *In vitro*	Inhibits the growth of F. nucleatum and P. gingivalis in both planktonic cultures and biofilms, demonstrating antibacterial effects	The physicochemical properties of mHA/CS scaffolds were examined by Fourier-transform infrared (FT-IR) spectroscopy, transmission electron microscopy (TEM), and Brunauer–Emmett–Teller (BET) analysis	Inhibits the growth of periodontal pathogens and promotes the formation of bone and cementum-like tissue
PLA	Zhang Y. et al. (2023)	*In vivo* and *In vitro*	Promotes cellular proliferation and differentiation	Proposing a biomimetic bone tissue engineering strategy enabled by a new type of Janus porous polylactic acid membrane (PLAM)	Promotes angiogenesis of human umbilical vein endothelial cells (HUVECs) and periodontal regeneration
PLGA	Ouyang et al. (2025)	*In vivo* and *In vitro*	Reduces levels of pro-inflammatory cytokines and elevates levels of anti-inflammatory cytokines in the gingival sulcus of rats with periodontitis	Synthesizing biodegradable microspheres (AZM@PLGA-SF) for sustained AZM release	Locally ameliorates periodontal inflammation and facilitates periodontal tissue regeneration
	Guo et al. (2024)	*In vivo* and *In vitro*	Promotes osteogenic differentiation of BMSCs	An infection-sensitive scaffold prepared by layer-by-layer assembly of Feraheme-like superparamagnetic iron oxide nanoparticles (SPIONs) on the surface of a three-dimensional-printed polylactic-co-glycolic acid (PLGA) scaffold	Achieves superior periodontal regeneration
Alginate	Suo et al. (2023)	*In vitro*	The scaffold had good biocompatibility and could promote the proliferation of hPDLCs	Constructing a carbon nanotube/chitosan/sodium alginate (CNT/CS/AL) ternary composite hydrogel and then preparing a porous scaffold by 3D printing technology	Promotes periodontal regeneration

Natural polymers, particularly chitosan, serve as a diverse range of drug carriers due to their degradation capabilities. Non-toxicity and high biocompatibility are additional specific properties that make chitosan an ideal candidate for drug delivery systems. Chitosan can be modified into various formulations tailored to specific functions and modes of administration. Furthermore, the protonated amino groups present on D-glucosamine in the chitosan structure enable it to adhere to negatively charged mucus layers through electrostatic interactions, allowing penetration into deeper epithelial layers (Aguilar et al., 2019; Bakshi et al., 2020; Qin and Li, 2020). Due to its mucoadhesive properties, chitosan has been utilized as a vehicle for administering drugs via nasal, ocular, buccal, and pulmonary routes (Bernkop-Schnürch and Dünnhaupt, 2012; Khutoryanskiy, 2011; Singh et al., 2023). However, the insolubility of chitosan in physiological environments poses a challenge for effective drug delivery; this limitation can be addressed through chemical modifications such as carboxymethylation, acetylation, thiolation, and quaternization (Chen K. et al., 2017; Wang W. et al., 2020).

#### 3.2.2 Polylactic acid

Polylactic acid (PLA) serves as an ideal polymer scaffold for dental, oral, and craniofacial tissue engineering owing to its hydrolytic degradation into lactic acid, a natural human metabolite (Diaz-Gomez et al., 2020). To enhance the scaffold performance, PLA can be combined with durable polymers, such as PEEK, via selective laser sintering to improve bioactivity, biocompatibility, and cytocompatibility (Feng et al., 2018). Additionally, blending PLA with PCL through 3D electrospinning enhances mechanical properties and promotes bioactivity and osteogenic differentiation (Yao et al., 2017). PLA scaffolds with stromal-derived factor-1 offer sustained release and promote endothelial cell proliferation and neovascularization, thus underscoring the potential of 3D-printed PLA constructs for bone regeneration (Singhvi et al., 2019). A novel biomimetic strategy employs Janus-structured porous PLA membranes (PLAMs) created via unidirectional evaporation-induced pore formation and self-assembled bioactive metal-phenolic network (MPN) nanointerfaces. Through the use of PLGA microspheres loaded with IL-4 (IL-4-PLGA), the composite hydrogel scaffold facilitated the transition of macrophages from the M1 phenotype to the M2 phenotype during the early induction phase. Additionally, it demonstrated a sustained anti-inflammatory effect mediated by PLGA microspheres containing kartogenin (KGN-PLGA) (Cheng et al., 2022). PLA serves as a carrier for doxycycline (Doxy) and nano-hydroxyapatite (nano-HAP), facilitating the gradual release of these substances through its resorption process. Consequently, it is anticipated that PLA will exhibit limited antibacterial properties. The enhanced antibacterial effect observed in the tested nanofibers, which were loaded with half the concentration of Doxy (5.04 µg) compared to the Doxy micro-compresses (10 µg), was evidenced by the larger diameters of the inhibition zones (Andrei et al., 2022). *In vitro*, MPN modification significantly reduced pro-inflammatory macrophage polarization in murine bone marrow-derived macrophages, stimulated angiogenesis in human umbilical vein endothelial cells, and enhanced the adhesion, migration, and osteogenic differentiation of human periodontal ligament stem cells. *In vivo*, PLAM-MPN implantation significantly accelerates bone repair in rat periodontal defects (Zhang Y. et al., 2023). This bioactive nanointerface within a Janus porous membrane has diverse capabilities to control cell physiology and promote bone regeneration, showing promise as a membrane for clinical applications in GTR and GBR.

#### 3.2.3 Poly (lactic-co-glycolic acid)

Poly (lactic-co-glycolic acid) (PLGA), a copolymer of lactic and glycolic acids, is widely used as a synthetic biomaterial for drug and growth factor delivery and as a barrier in GTR/GBR. Its degradation rate can be adjusted by varying the lactic-to-glycolic acid ratio, owing to the differing hydrophilicities of the monomers (Washington et al., 2017). *In vitro* tissue engineering studies have compared various PGA-based polymers, such as PGA–PLA, PGA–PCL, and PGA–poly-4-hydroxybutyrate (P4HB). Among these, the PGA–PLA and PGA–P4HB scaffolds showed superior tissue formation compared to PGA–PCL, likely due to an optimal balance between scaffold degradation and tissue formation, thereby preserving mechanical integrity (Generali et al., 2017). PLGA’s excellent biocompatibility and tunable degradation properties make it versatile for periodontal regeneration applications, including barrier membranes, bone grafts, and delivery systems (Sun et al., 2017). Tetracycline, a widely utilized antibacterial agent, was effectively incorporated into PLGA membranes (Owen et al., 2010). The release profiles demonstrated an initial burst phase during the first week, followed by a sustained drug release over a duration of 14 days. This pattern is advantageous for achieving high antibiotic concentrations in the early stages while ensuring an adequate dose throughout the entire healing process. Notably, the incorporation of tetracycline did not alter the morphology of PDLSCs seeded onto the membranes. Kim et al. (2007) investigated the *in vivo* release profile of tetracycline-loaded PLGA membranes. The results indicated that sustained tetracycline release occurred with elevated concentrations during the initial 7 days; additionally, a greater extent of tissue attachment was observed compared to PLGA membranes alone. Azithromycin (AZM) was encapsulated in PLGA microspheres (AZM@PLGA) using single emulsion solvent evaporation and then coated with silk fibroin (SF) via electrostatic adsorption to mitigate AZM’s initial burst release (Ouyang et al., 2025). AZM@PLGA-SF microspheres significantly reduced periodontal inflammation and restored periodontal tissue health *in vivo*. The mechanically formulated microspheres modulated the inflammatory microenvironment in periodontal tissues by reducing pro-inflammatory cytokines (tumor necrosis factor-α, IL-6, interferon-γ, IL-2, and IL-17A) in gingival crevicular fluid while increasing anti-inflammatory cytokines (IL-4 and IL-10). In a rat model of periodontal defects, the superparamagnetic iron oxide nanoparticle (SPION)/PLGA scaffold enhanced IL-10 production and decreased NLRP3 and IL-1β release following *Porphyromonas gingivalis* infection, resulting in superior periodontal regeneration compared to the PLGA scaffold alone. This antibacterial SPION/PLGA scaffold exhibited anti-inflammatory and anti-adhesive properties, aiding in infection control and promoting periodontal tissue regeneration through immune modulation (Washington et al., 2017).

#### 3.2.4 Alginate

Alginate, a polysaccharide derived from brown algae, is used as a hydrogel in 3D bioprinting and tissue engineering because of its cost-effectiveness, wide compatibility, and similarity to proteins in the ECM. Alginate polymerization is rapid and typically occurs within seconds to minutes of exposure to divalent cations, allowing for various ion-doping options. The introduction of methacrylate groups enables hydrogel production through light-induced radical polymerization. However, the inherent structure of alginate lacks peptide-binding sites for cellular interactions, such as arginine–glycine–aspartate (RGD) and YIGSR peptides (Tolmacheva et al., 2024). Therefore, alginate is commonly combined with other cell-adhesive polymers, such as collagen, and functional polymers, such as agarose or hyaluronic acid, by incorporating an ECM-based protein or RGD peptide sequence to enhance cell adhesion and proliferation. The loading of the drug (spermidine) via dynamic bonding presents a promising avenue, offering a straightforward, cost-effective, and efficient strategy for the sustained release of pharmaceuticals while enhancing the viscoelastic properties of hydrogels (Zhang S. et al., 2023). The drug-loadable calcium alginate hydrogel system is a potential bone defect repair material for clinical dental applications (Chen L. et al., 2017). Bovine serum albumin (BSA) can be directly stored within the 3D pore network structure of hydrogels. However, the positively charged functional groups in BSA can interact with the carboxyl groups present in alginate, leading to the formation of BSA/alginate complexes. Consequently, the release of BSA from these hydrogels is sustained as hydrogel degradation progresses (Zhao et al., 2009). Calcium alginate hydrogels, thus, possess the ability to combine with drugs or proteins through various mechanisms, rendering them highly suitable for drug delivery applications. Collagen provides additional benefits, including low immunogenicity and the promotion of bone regeneration. The use of collagen matrices has been shown to influence osteoblast behavior in various hydrogels (Tharakan et al., 2022). To expedite gelation, post-bioprinting self-healing, and subsequent stabilization via secondary crosslinking, alginate was subjected to methacrylation and oxidation, followed by ionic crosslinking using an eggshell model (Jeon et al., 2022). To enhance alginate hydrogels for bone tissue engineering, inorganic particles, such as bioglass and kaolin, or organic materials, such as cells, drugs, and human allograft tissue, can be integrated (Bhattacharyya et al., 2024; Bhattacharyya et al., 2023; Gharacheh and Guvendiren, 2022). The CS/alginate hydrogel exhibits certain unavoidable limitations, including a high degradation rate and low mechanical strength (Hu et al., 2019; Zupančič et al., 2019). Furthermore, research indicates that the incorporation of carbon nanotubes (CNTs) into the composite scaffold enhances its mechanical properties, potentially making it suitable for periodontal tissue regeneration (Carletti et al., 2011). Consequently, compared to bare hydrogel CS/AL composites, CNT/CS/AL demonstrates superior mechanical and antibacterial properties. Studies have indicated that an RGD-coupled alginate scaffold promotes the differentiation of oral MSCs toward an osteoblast lineage both *in vitro* and *in vivo*, as evidenced by the expression of the osteogenic markers Runx2, ALP, and osteocalcin. In summary, these findings represent the first evidence that MSCs from the orofacial tissue, when encapsulated in RGD-modified alginate scaffolds, have the potential for craniofacial bone regeneration (Gharacheh and Guvendiren, 2022). The use of an hPDLSC-based medium enhanced the osteogenic differentiation of hPDLSCs in the Alg + Fib + hPL construct, offering a promising xeno-free strategy for delivering hPDLSCs to boost dental, craniofacial, and orthopedic regenerations (Qiu et al., 2022).

### 3.3 Hydrogel

**FIGURE 4:**
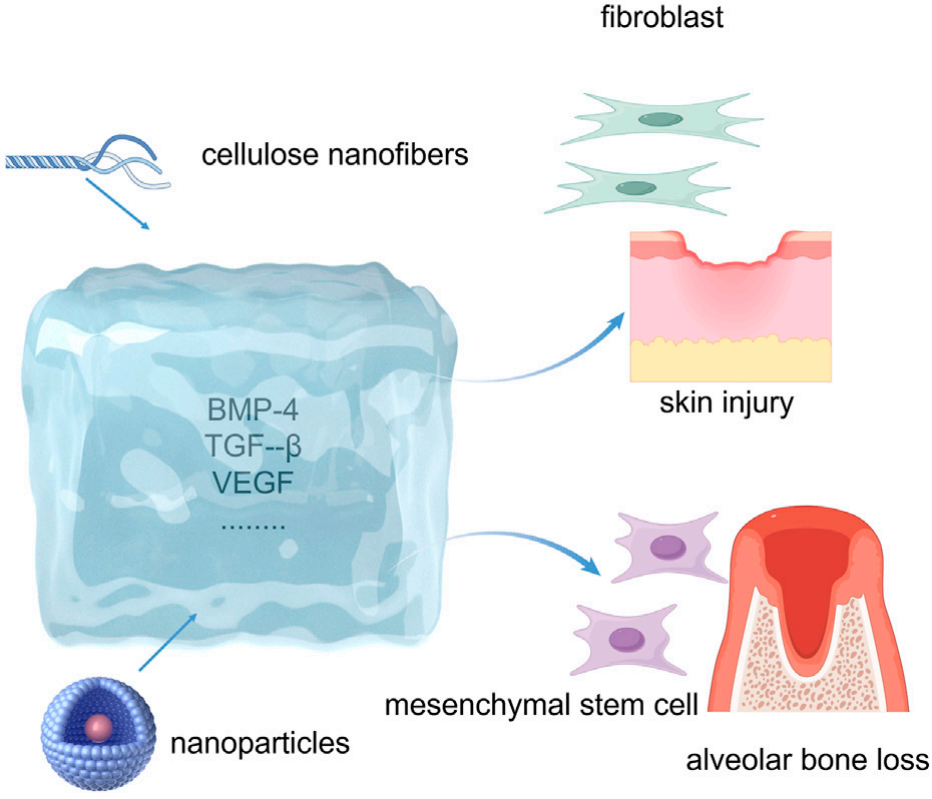
Hydrogels for periodontal tissue regeneration.

Periodontal tissue regeneration is complex. Hydrogels exhibit significant potential for clinical applications in this domain by closely mimicking natural ECM and biological tissues (Sánchez-Cid et al., 2022) (Figure 4). These polymeric materials possess a three-dimensional network of physically or chemically linked polymer chains that enables them to absorb and retain substantial amounts of water while maintaining their solid structure. The high water content of hydrogels contributes to their softness and biocompatibility and creates an optimal environment for cell adhesion, proliferation, differentiation, and growth (Radulescu et al., 2022). Hydrogels have been extensively investigated as scaffolds and/or drug delivery systems because of their ability to integrate cells into their structures and concurrently degrade with new tissue formation (Santos et al., 2023b). Xu et al. (2019) developed an injectable thermosensitive hydrogel that facilitates the continuous release of aspirin and erythropoietin, thereby promoting the healing of inflammation and the repair of alveolar bone. Within the first 3 days of the drug release trial, 86.6% and 69.4% of aspirin and erythropoietin were released, respectively. The rapid release rate of aspirin is particularly beneficial during the early stages of periodontitis treatment, where prompt control of inflammation is essential. The thermosensitive hydrogel designed by Liu et al. (2022) used polyethylene glycol diacrylate as the base scaffold and incorporated additional reactive oxygen species (ROS)-scavenging effects to enhance its functionality. By adding dithiothreitol, a reducing sugar alcohol, this hydrogel also demonstrated oxidative stress-reducing effects on cells, thus providing protection for periodontal cells against inflammatory damage. Furthermore, by incorporating various bioactive molecules, hydrogels can induce crucial cellular processes, such as migration, proliferation, differentiation, vascularization, and mineralization during periodontal regeneration (Gopalakrishna et al., 2023; Ranch et al., 2021; Swain et al., 2019). The hydrogel matrix can be enhanced through the incorporation of organic or inorganic nanomaterials. This addition improves surface reactivity, mechanical properties, and the release kinetics of loaded bioactive agents (Irawan et al., 2018; Radulescu et al., 2022). Additionally, hydrogels offer excellent biocompatibility, water retention, controlled release, and support for cellular interactions, making them promising candidates for periodontal tissue engineering (Zhang W. et al., 2021). The mechanical strength of hydrogels represents a significant limiting factor in their application for periodontal tissue engineering. Striking an optimal balance between mechanical strength and injectability poses considerable challenges, as enhancing the mechanical properties frequently leads to a reduction in injectability (Sun et al., 2024) (Table 5).

**TABLE 5:** Hydrogel materials used in periodontal tissue regeneration.

Material	Reference	*In vivo*/*in vitro*	Biocompatibility	Method	Result
GelMA	Yang et al. (2025)	*In vivo*	Promotes collagen deposition, cell migration, and wound healing	CuSNPs were first precipitated by chitosan (CS) and then grafted with methacrylic anhydride (MA) to form CuSNP@CS-MA, which was photo-crosslinked with GelMA to synthesize hybrid hydrogels (GelMA/CuSNP)	Accelerates alveolar osteogenesis and vascular genesis
	Wang Y. et al. (2022)	*In vivo*	Promotes cell proliferation, migration, and tube formation, and has therapeutic effects on diabetic wound healing	By activating the HIF-1α/VEGFA signaling pathway, local angiogenesis is promoted	Promotes wound healing by enhancing local blood supply and angiogenesis
	Hu et al. (2024)	*In vivo*	Promote cell proliferation and migration	Engineering an interconnected porous bovine serum albumin methacryloyl (BSAMA: B, as a drug carrier and antioxidant)/gelatin methacryloyl (GelMA: G, as a biocompatible collagen-like component)-based cryogel with L-arginine (Arg) loaded as an angiogenic molecule	Promotes vessel formation and collagen deposition
Polysaccharide hydrogel	, Frasca et al. (2017)	*In vivo*	A migration of labeled adipose-derived stromal cells from the center to the periphery of the hydrogel was associated with a better bone tissue regeneration process	Syngeneic mesenchymal stem cells (5 × 10 5) and macroporous biphasic calcium phosphate ceramic granules (Calciresorb C35 ®, Ceraver) or porous pullulan/dextran-based hydrogel scaffolds were implanted alone or combined in a drilled-hole bone defect in rats	Enhances both bone healing and neovascularization

#### 3.3.1 Gelatin methacryloyl (GelMA)

Gelatin methacryloyl (GelMA), akin to the native ECM, features multiple RGD sequences for cell adhesion and MMP-degradable motifs for cell remodeling (Klotz et al., 2016). As a photo-crosslinked hydrogel, GelMA undergoes radical polymerization initiated by light, eliminating the need for potentially harmful chemical cross-linkers (Klotz et al., 2016). Despite its remarkable characteristics, it is primarily constrained by its limited mechanical strength, as certain tissue types inherently exhibit high mechanical stiffness. Although the application of elevated GelMA concentrations can enhance mechanical strength, this improvement comes at the expense of porosity, degradability, and three-dimensional (3D) cell attachment (Sakr et al., 2022). The tunable mechanical properties and favorable biological traits of GelMA make it a versatile scaffold for diverse biomedical applications (Sakr et al., 2022). Studies have shown that GelMA-based scaffolds enhance cell viability and promote osteogenic/odontogenic differentiation of dental stem cells, including dental pulp stem cells (DPSCs), PDLSCs, and stem cells from the apical papilla (SCAPs) (Zhu et al., 2023). In addition to serving as a cell delivery carrier in bioprinting, GelMA facilitates drug and growth factor delivery owing to its degradability. FT-IR spectroscopy confirmed the successful incorporation of irradiated chlorpromazine (CPZ) into the hydrogel matrix. The release profiles of chlorpromazine demonstrated sustained release exclusively in hydrogels containing 1 mg/mL of CPZ. Furthermore, the hydrogel exhibited significant antimicrobial activity against MRSA bacteria compared to penicillin. These findings underscore the potential of CPZ loaded during the photopolymerization process within hydrogels as an effective antimicrobial agent with sustained release properties, rendering it suitable for combating resistant bacterial strains (Tozar et al., 2024). Park et al. (2020) developed a bioink by conjugating BMP peptides to GelMA and found that the BMP-mimicking peptide remained in the bioprinted structure for over 3 weeks and significantly increased odontogenic gene expression in hDPSCs. Pan et al. (2020) evaluated a GelMA hydrogel containing human PDLSCs both *in vitro* and *in vivo*, noting enhanced PDLSC proliferation and differentiation within the hydrogel, along with new bone formation in rat alveolar defects treated with hydrogels. GelMA hydrogels have also been improved by using nano-hydroxyapatite (Chen X. et al., 2016). Human PDLSC constructs significantly enhanced osteogenic differentiation, resulting in increased mineralized tissue formation *in vivo* in a mouse model. GelMA has been combined with materials, such as polyethylene glycol diacrylate (Ma et al., 2017). In diabetic mice, GelMA hydrogels with VH-EVs (Gel-VH-EVs) effectively promoted wound healing by enhancing local blood supply and angiogenesis, potentially by activating the HIF-1α/vascular endothelial growth factor (VEGF)A signaling pathway (Wang Y. et al., 2022). Additionally, GelMA/CuSNP hydrogels accelerate alveolar osteogenesis and vascular genesis, successfully treating periodontitis within 4 weeks in a rat model (Yang et al., 2025).

#### 3.3.2 Polysaccharide hydrogels

Polysaccharide hydrogels have emerged as pivotal in bone tissue engineering and regenerative medicine primarily because of their efficacy in enhancing bone repair via cellular and drug delivery. They provide a hydrophilic 3D matrix that enhances osteocyte viability and promotes new bone formation. These hydrogels are extensively used in tissue engineering and wound healing owing to their superior biocompatibility, controlled degradation, and versatile structure (Gonzalez-Fernandez et al., 2022). Their porous polymeric frameworks and high hydration levels emulate the native ECM, supporting cellular proliferation, differentiation, and tissue repair. Furthermore, their biodegradable nature, customizable chemistry, low toxicity, and minimal immune response render them ideal candidates for sustained drug release. In scaffold-based applications, polysaccharide hydrogels induce a foreign body response similar to that of soft tissues, closely mimicking the natural ECM (Taghipour et al., 2020).

Polysaccharide hydrogel scaffolds leverage several key mechanisms to facilitate bone regeneration. First, the hydrogel exhibits low immunogenicity and contains multiple functional groups in its backbone that serve as attachment sites, preventing the displacement of bone cells and therapeutics by blood flow, thereby enabling bone cell proliferation at the fracture site (Koons et al., 2021). Second, the scaffold incorporates MSCs and various osteoinductive proteins, including BMP-2, transforming growth factor-β (TGF-β), and VEGF (Aravamudhan et al., 2013; Gugliandolo et al., 2021; Xu X. et al., 2012). Third, the polysaccharide hydrogel surface is highly microporous, which enhances vascularization and stimulates innate osteogenic processes (Gonzalez-Fernandez et al., 2022). Scaffold-based polysaccharide hydrogels possess porous structures that promote bone regeneration. Studies have demonstrated the remarkable mechanical properties and bone-regenerative capacity of polysaccharide hydrogels. Kolk et al. (2012) integrated regenerated cellulose nanofibers into a chitosan-based polysaccharide hydrogel that enhanced the proliferation and differentiation of osteoblasts. Polysaccharide-based hydrogels were doubly integrated with hydroxyapatite (HAp) nanoparticles and calcium carbonate microspheres (CMs) under physiological conditions. Furthermore, the composite gel scaffolds exhibited favorable sustained drug release and antibacterial properties, as confirmed by calculations of drug release and evaluations of antibacterial activity (Ren et al., 2018). Overall, microporous polysaccharide hydrogels offer excellent biocompatibility, biodegradability, self-repair capabilities, and injectability, making them promising candidates for bone tissue engineering applications.

### 3.4 Decellularized extracellular matrix

#### 3.4.1 dECM hydrogels

Decellularized extracellular matrix (dECM) hydrogels are increasingly used in tissue engineering, particularly for *in vitro* organoid development. This intricate macromolecular framework plays a vital role in modulating cellular adhesion, movement, specialization, and functional activity, thereby influencing tissue growth and maintenance (Zhang H. et al., 2021). Among commercial ECM products, Matrigel®—derived from murine tumor secretions—remains widely used due to its rich composition of basement membrane components, including laminin, type IV collagen, entactin, and heparan sulfate. Various tissue sources, such as human, porcine, bovine, and murine tissues, have been processed to yield dECM using mechanical, chemical, or enzymatic decellularization methods (Crapo et al., 2011). The resulting dECM can be gelled by temperature modulation, ionic strength adjustment, pH variation, or chemical crosslinking. As biomimetic materials, dECM-based constructs offer a tissue-specific microenvironment that promotes specialized cell functions and enhances natural regeneration (Gattazzo et al., 2014). Decellularization effectively eliminates cellular material and immunogenic components while retaining key structural proteins (e.g., collagen, elastin, and fibronectin) and macromolecules (e.g., proteoglycans and glycosaminoglycans) (Keane et al., 2015).

The dECM is extensively utilized in regenerative medicine as a scaffold material, owing to its exceptional biological activity and excellent biocompatibility. Advanced manufacturing techniques, such as 3D printing and electrospinning, have been employed to produce materials/scaffolds based on dECM that can replicate the distinct characteristics of periodontal tissues (Schmidt-Schultz and Schultz, 2005). Multiple strategies have been developed to enhance the mechanical strength of the dECM (Jiang et al., 2021). These include functionalized scaffolds embedded with cells that facilitate the harvesting of scaffold-supported dECM through a process of decellularization and crosslinked soluble dECM that can be utilized to form injectable hydrogels for periodontal tissue repair (Liang et al., 2023). This expansion of dECM applications has facilitated the clinical translation of dECM-based materials for periodontal regeneration. Consequently, the dECM serves as a crucial source of bioactive material to initiate the repair of periodontal tissue and regeneration of the periodontium (Papadimitropoulos et al., 2015). For instance, bone dECM serves as a reservoir of pro-inflammatory cytokines, growth factors from the TGF-β family, various bone morphogenetic proteins (BMPs), and angiogenic growth factors, such as VEGF, thereby inducing osteoinduction by controlling different stages of bone regeneration (Wang S. et al., 2022). The immunomodulatory activity of dECM is robust, leading to a reduction in the secretion of pro-inflammatory factors by M1 macrophages and a decrease in local inflammation in Sprague–Dawley rats. In a critical-size model of periodontal defects, the bioprinted module significantly boosted the regeneration of hybrid periodontal tissues in beagles, particularly the anchoring structures of the bone–ligament interface, well-aligned periodontal fibers, and highly mineralized alveolar bone (Yang et al., 2023).

The urinary bladder matrix and SIS are dECM biomimetic materials endorsed by the FDA to create regenerative biomimetic materials for damaged skin, muscle, and gastrointestinal tissues (Brown-Etris et al., 2019). SIS, a commonly used decellularized tissue, shows promise for soft tissue repair but requires enhancement for bone tissue regeneration. Gou et al. (2019) utilized pig SIS as a collagen membrane to guide tissue regeneration. In a rat cranial defect model, SIS dECM facilitated bone tissue regeneration, resulting in an increased bone volume fraction and tissue regeneration, suggesting the potential for bone regeneration in periodontal defects.

#### 3.4.2 Hard tissue-derived dECM

**FIGURE 5:**
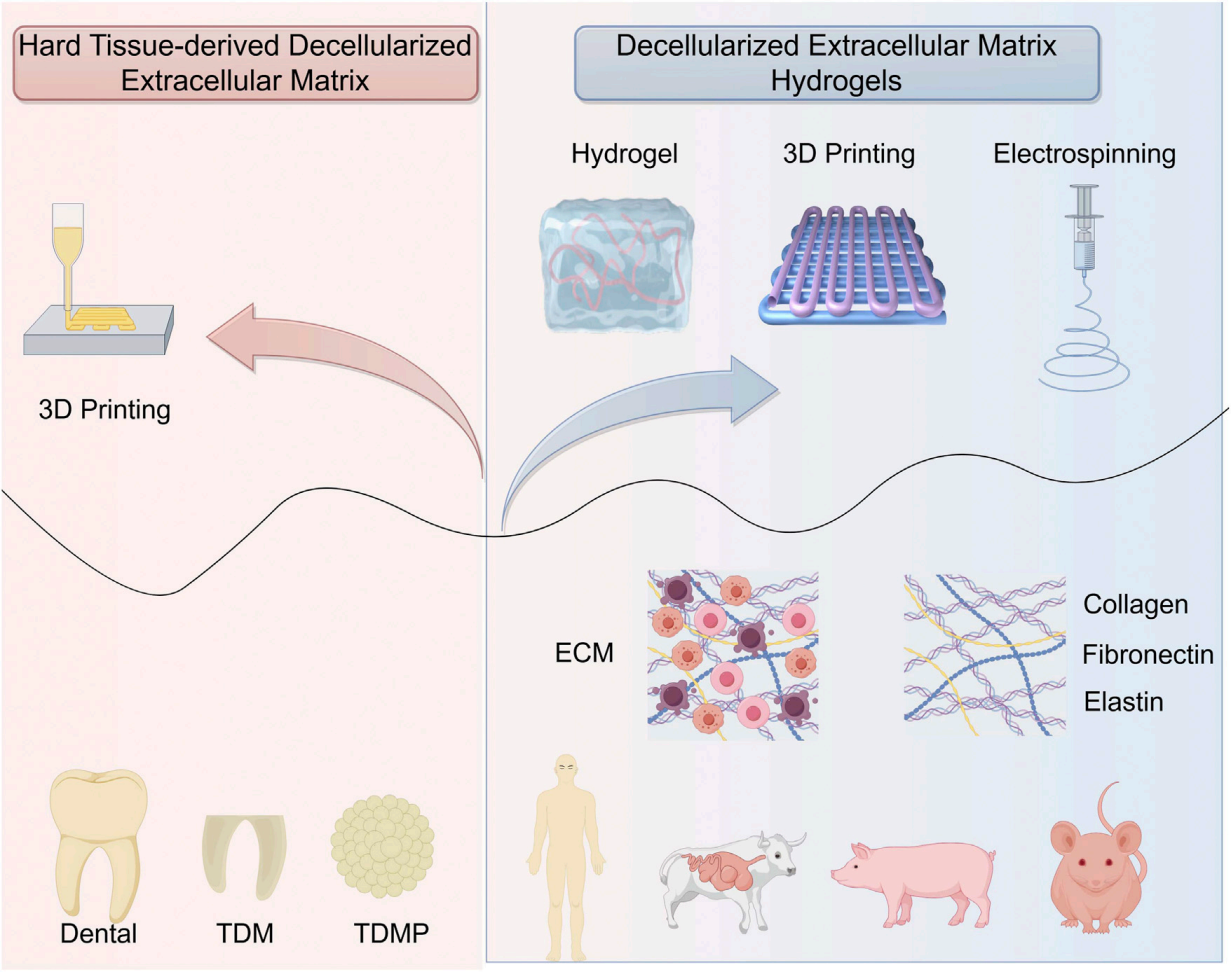
Decellularized extracellular matrix for periodontal tissue regeneration.

Decellularized bone powder, a common periodontal tissue engineering material, is clinically used in the form of dECM granules (Figure 5). These granules are often combined with hydrogels and a guiding tissue regeneration membrane to enhance periodontal regeneration (Suzuki et al., 2023). Datta et al. (2021) used decellularized bone granules within a chitosan-based hydrogel containing human amniotic mesenchymal stem cells (hAMSCs) to stimulate bone regeneration. Intrinsic growth factors present in decellularized bone granules promote robust cellular viability, proliferation, and osteogenic differentiation of human amnion-derived stem cells (Datta et al., 2021). This biomaterial shows promise for application in periodontal tissue engineering because of its ability to effectively preserve the intricate ECM components found in native tissue, offering optimal cues for the regeneration and repair of damaged periodontal tissue. Son et al. (2019) treated tooth slices from human molars with sodium dodecyl sulfate and Triton X-100, effectively removing nuclear components while preserving the structure and composition (Son et al., 2019). The T-dECMs supported PDLSC regeneration near the cementum by expressing cementum- and PDL-related genes. These findings demonstrate that dentin dECM enhances MSC proliferation and differentiation and show promise for periodontal regeneration in tissue engineering. Dental-derived dECM has been used successfully to promote dental tissue repair (Jeon et al., 2022; Liang J. et al., 2020). The development of soluble dECM, 3D printing, and other advanced fabrication techniques has helped improve the fidelity and strength of dECM-based 3D scaffolds (Park, 2019), focusing on the spatial management in the defect area (Kim et al., 2020; Varoni et al., 2018; Yang et al., 2023). Meanwhile, due to the abundance of certain functional groups in dECM materials—such as hydroxyl, acyl, peptide bonds, and hydrogen bonds—these materials facilitate drug loading through crosslinking reactions (Ding et al., 2023). Notably, this includes amide crosslinking and interactions between aldehyde groups and the amine groups of lysine or hydroxylysine (Gou et al., 2019).

The treated dentin matrix (TDM) exhibits strong odontogenic/osteogenic potential and has been demonstrated to regenerate periodontal ligament-like tissues, including perforating fiber-like tissues, in a beagle canine alveolar socket orthotopic transplantation model. Meanwhile, TDM has the ability to provide sustained drug release, and xenogeneic TDM equipped with the immunomodulatory drug rosiglitazone can achieve a periodontal tissue regeneration effect comparable to that of homogeneous TDM (Lan et al., 2021). Surprisingly, TDM could be ground into TDM particles and combined with PCL, which could be used to fabricate personalized biological roots using low-temperature deposition manufacturing 3D printing technology and regenerate periodontal ligament-like tissue in a beagle canine alveolar socket orthotopic transplantation model. Owing to the superior personalized matching capability of 3D-printed scaffolds, the TDM-PCL scaffold enables the replication of the “immediate implantation” process in the *in situ* transplantation experiment of the alveolar socket. Therefore, TDM is a type of dECM that plays an important role in periodontal tissue regeneration (Huang et al., 2023) (Table 6).

**TABLE 6:** dECM materials used in periodontal tissue regeneration.

Material	Reference	*In vivo*/*in vitro*	Biocompatibility	Method	Result
Decellularized extracellular matrix hydrogel	Ivanov et al. (2023)	*In vitro*	Facilitates the recruitment of populations of resident cells into damaged tissues and stimulates their proliferation and differentiation	Evaluating the effect of the exogenous components of the ECM on the differentiation of periodontal ligament stem cells (PDLSCs) cultured with dECM in a 3D collagen I hydrogel	Promotes the dECM most effectively and induces the differentiation of PDLSCs into osteoblast-like and odontoblast-like cells
	Dziedzic et al. (2021)	*In vivo*	Stimulates ECM deposition and cell differentiation, and guarantees the preparation of cell sheets for clinical transplantation	Applying the decellularized amnion infiltrated by adipose mesenchymal stem cells (ADSCs) with mineralized extracellular matrix to a rat periodontal root bifurcation lesion model	Promotes periodontal regeneration
	Huang et al. (2021)	*In vitro*	Facilitates the proliferation of PDLSCs and differentially affects their differentiation	The proliferative capacity of PDLSCs was evaluated using the PicoGreen Assay Kit. The expressions of alkaline phosphatase (ALP), runt-related transcription factor-2 (RUNX2), osteocalcin (OCN), collagen I (COL1), periostin (POSTN), and cementum protein 1 (CEMP1) were detected by qRT-PCR and Western blotting	Facilitates osteogenic differentiation and periodontal ligament differentiation of PDLSCs
Hard tissue-derived decellularized extracellular matrix	Lan et al. (2021)	*In vivo*/*in vitro*	Promotes cell adhesion and proliferation	TDM can be ground into TDM particles and combined with PCL	Promotes periodontal tissue regeneration

### 3.5 Autologous materials as regenerative substitutes for periodontal tissue regeneration

Numerous regenerative techniques have been developed for the treatment of periodontitis. However, current treatment modalities, whether used alone or in combination, face challenges in achieving genuine regeneration, particularly for complex periodontal defects. Consequently, there is a need to identify an optimal material that can facilitate periodontal regeneration with enhanced potential for new attachments and reduced complications. Clinical trials in regenerative medicine have explored the use of allografts, xenografts, alloplasts, or their combinations, based on their documented regenerative capabilities (Liang et al., 2021). Nevertheless, these bone graft materials are hindered by the significant drawback of eliciting foreign body reactions or adverse immune responses, which limits their efficacy in treating periodontal defects. Due to these constraints, clinicians favor autogenous biomimetic materials that promote tissue regeneration with minimal immunogenicity upon introduction into the body. One such biomaterial is the fibrin matrix known as platelet-rich fibrin (PRF), which was initially described by Choukroun. PRF, a second-generation clot derived from a patient’s own blood through centrifugation, transforms into a robust three-dimensional fibrin scaffold containing platelets, leukocytes, and various growth factors (such as platelet-derived growth factor (PDGF), VEGF, TGF-β, insulin-like growth factor 1 and 2 (IGF-1/IGF-2), and epidermal growth factor (EGF)), crucial for tissue regeneration (Christgau et al., 2006; Dohan et al., 2006). The findings highlight the potential of antibiotic-loaded PRF as an effective localized drug delivery system exhibiting robust antibacterial properties (Kobayashi et al., 2016). For patients not undergoing systemic antibiotic therapy, the optimal strategy involves incorporating the antibiotic into PRF or i-PRF only after centrifugation (Tan et al., 2019). Compared to other carriers, such as collagen, PRF consistently demonstrates superior antibacterial effects and enhanced drug-loading capacity (Niemczyk et al., 2025). Biological proteins induce the differentiation of periodontal ligament cells into osteoblasts, thereby promoting bone regeneration. In a clinical trial, Serroni et al. (2022) found that leukocyte platelet-rich fibrin (L-PRF) enhanced the degree II furcation management when combined with autogenous bone grafts compared to autogenous bone grafts and open-flap debridement alone. In a randomized trial, Rexhepi et al. (2021) demonstrated that combining an inorganic bovine bone graft with L-PRF was as effective as combining it with a collagen membrane (CM) in managing unfavorable intrabony defects. Various PRF variants have emerged over time owing to different centrifugation protocols aimed at enhancing growth factor release. Advanced platelet-rich fibrin (A-PRF) developed by Choukroun in 2014 utilizes a low centrifugal speed (150 rpm, 14 min) to achieve a more uniform distribution of neutrophils and an unbound fibrin matrix. Neutrophils in PRF contribute to tissue regeneration by guiding monocyte phagocytosis and producing proteases such as MMP9 involved in wound healing (Fujioka-Kobayashi et al., 2017). Moreover, PRF application in periodontal intrabony treatment led to a reduced pocket probing depth and improved relative attachment levels. Lei et al. (2020) conducted a study utilizing A-PRF and concentrated growth factor (CGF) to treat periodontal defects and demonstrated a consistent and sustained release of growth factors for 2 weeks. The authors noted that this combined approach exhibited comparable efficacy in regenerating periodontal bone and enhanced GTR results in intrabony defects (Lei et al., 2020). Furthermore, the combination of A-PRF+ with xenografts or alloplastic materials may enhance periodontal tissue regeneration, facilitate bone augmentation, and contribute to improved implant stability (Chmielewski et al., 2025). One study utilized human gingival fibroblasts, whereas the other employed human periodontal ligament cells (Miron et al., 2017). Blood samples for these experiments were collected exclusively from human participants (Zheng et al., 2020). One study demonstrated that both PRP and i-PRF exhibited excellent cell viability and biocompatibility (Farshidfar et al., 2025).

## 4 Physical properties

Successful tissue regeneration requires scaffolds with specific mechanical stability and biodegradability, appropriate dimensions, surface roughness, and porosity to create a suitable microenvironment that supports effective cell–cell interactions, cell migration, proliferation, and differentiation. Biomimetic materials’ physical properties, such as pore size, porosity, particle size, and mechanical strength, play a crucial role in influencing cell attachment and proliferation (Bharadwaz and Jayasuriya, 2020).

### 4.1 Fabrication for scaffold

Periodontal tissues, which include the gingiva, periodontal ligament, cementum, and alveolar bone, play a critical role in supporting and anchoring teeth within the jawbone. However, the regeneration of the complex structural architecture of periodontal tissues continues to pose a significant challenge. Given the complexity of periodontal tissues and the need to promote coordinated and orderly tissue regeneration, developing hierarchical scaffolds that can simulate various periodontal tissues is an attractive strategy for achieving more synchronous tissue regeneration. Hierarchical scaffolds fabricated by various techniques such as 3D printing/bioprinting (Sufaru et al., 2022), electrospinning (Santos et al., 2023a), and hydrogel synthesis (Santos et al., 2023b) have different designs and compositions and have shown promising results in both *in vitro* and *in vivo* studies (Table 7).

**TABLE 7:** Scaffold fabrication for periodontal tissue regeneration.

Material	Reference	Type	Method	Result
Chitosan/β-glycerophosphate (CTS) hydrogels	Zang et al. (2019)	Hydrogel synthesis	CTS hydrogel loaded with BMP-7 and the antibiotic ornidazole	Demonstrates the enhancement of new alveolar bone and cementum formation
CTS hydrogel	Ammar et al. (2018)	Hydrogel synthesis	CTS hydrogels with freeze-dried platelet concentrate	Exhibits a sustained release of TGF-β1 and PDGF over 2 weeks, and significantly increased PDLSC viability
PEG/PCL/COL1	Sun et al. (2016)	Electrospinning	PCL/COL I electrospun scaffolds with PEG and calcium phosphate nanoparticles (NPs) containing recombinant human CMP1	Promotes the formation of cementum-like tissue instead of new bone formation in a rat calvarial defect
PCL/PEG	Jiang et al. (2015)	Electrospinning	The scaffold was fabricated by electrospinning and stacking 30 layers of fibrous mats in a CTS solution	Exhibits higher expression levels of POSTN, an increased collagen I/III ratio, and the formation of oriented PDL-like fibers in the regenerated periodontium
PCL/gelatin scaffold with HAp NPs (PGH)	Liu J. et al. (2021)	3D printing/bioprinting	PCL/PGH fabricated via extrusion printing	The biphasic scaffold exhibits significantly greater new bone formation compared to the PGH scaffold alone
PCL/HA	Pilipchuk et al. (2016)	3D printing/bioprinting	PCL/HAp scaffold, fabricated via selective laser sintering and seeded with BMP7 expressing	Increases the thickness of oriented collagen fibers, improves cell alignment, and greater nuclear elongation perpendicular to the dentin surface

To address this, it is essential to carefully design and fabricate suitable scaffolds based on specific clinical requirements in order to fully realize their functional potential. Electrospun nanofibers exhibit high porosity and large surface area, effectively mimicking the natural extracellular matrix and thereby modulating cell attachment, migration, proliferation, and differentiation. Recently, various electrospun nanofibrous membranes have been developed with antibacterial, anti-inflammatory, and osteogenic properties, demonstrating promising potential for periodontal regeneration (Santos et al., 2023a). The 3D bioprinting technology, particularly when utilizing biomaterials embedded with living cells and bioactive molecules, offers precise and controllable regulation of key structural properties such as porosity, connectivity, pore size, and permeability. This additive manufacturing approach constructs complex 3D architectures by systematically depositing cell-laden hydrogels or other biomaterials layer by layer. Although various 3D bioprinting techniques have been developed, each possesses distinct advantages and limitations (Dey and Ozbolat, 2020). Therefore, selecting an appropriate 3D bioprinting method that can accurately mimic the intricate structure and functional properties of native periodontal tissues is crucial for reconstructing a conducive regenerative microenvironment and promoting effective tissue regeneration (Shapira and Dvir, 2021).

### 4.2 Cell adhesion

Focal cell adhesion to various scaffold surfaces is crucial for initiating signaling cascades that further promote cell proliferation and differentiation (Lee et al., 2004). Therefore, precise regulation of cell–cell and cell–scaffold interactions can achieve multiple objectives in multifunctional tissue engineering. Biological material scaffolds can provide mechanical support and regulate cell adhesion and motility. Additionally, growth factors delivered by these scaffolds can modulate cellular behavior (Table 8).

**TABLE 8:** Cell adhesion in periodontal tissue regeneration.

Material	Reference	Cell type	Method	Result
ECM derived from human urine-derived stem cells (hUSCs)	Xiong et al. (2019)	hPDLSCs	Both hUSCs and hPDLSCs were seeded on TCP and stimulated to produce the ECM	Promotes hPDLSC proliferation, spreading, and adhesion
Calcium silicates	Zhang X. et al. (2014)	hPDLSCs	Zirconium-modified calcium silicate (Ca_3_ZrSi_2_O_9_)-based ceramics	Promotes hPDLSC proliferation, and adhesion
Polyurethane	He et al. (2022)	Mesenchymal stem cells	A biodegradable, bilayer polyurethane fibrous membrane integrated with the bioactive molecule dopamine	Effectively promotes cell adhesion and mesenchymal stem cell growth, and supports mineralization and antioxidant properties
PCL/PLA	Abdelaziz et al. (2021)	hPDLSCs	Electrospun nanofibrous scaffolds based on PLA or PCL polymers with silver and HA nanoparticles	Enhances cell adhesiveness
CS	Lee et al. (2016)	Human gingival fibroblasts	CS membrane with epigallocatechin-3-gallate (EGCG) grafted on the outer layer for bactericidal activity, and lovastatin was included in the middle layer for controlled release	Significantly enhances adhesion and proliferation of human gingival fibroblasts
GelMA/nHA	Chen X. et al. (2016)	hPDLSCs	Microgel arrays were fabricated by blending different weight ratios of GelMA and nHA	The GelMA/nHA microgels exhibit appropriate microarchitecture, mechanical strength, and surface roughness, thus enabling cell adhesion and proliferation

The ECM offers significant advantages in regulating cell adhesion. All cells interact with the ECM, which plays a crucial role in determining stem cell fate. Different types of extracellular matrices vary in composition and function (Watt and Huck, 2013). Moreover, upon cell binding to the ECM, intracellular signaling is regulated through cell surface adhesion receptors, among which the integrin family represents a major and essential class. Additionally, various protein domains within the ECM can dynamically bind and release growth factors, thereby synergizing with these factors to modulate cellular behavior (Hynes, 2009). Furthermore, ECM features such as micro-patterned islands, stiffness, physical topography, and deformability can promote distinct biological processes (Watt and Huck, 2013).

### 4.3 Porosity

The functional performance of implanted cells is largely determined by the pore size of the scaffold. Pore dimensions critically influence the diffusion of nutrients and oxygen, along with the removal of metabolic waste. Furthermore, pore size significantly affects cell adhesion, cell-to-cell interactions, and transmembrane cell migration—processes that vary according to the specific requirements of different tissue regeneration applications (Bružauskaitė et al., 2016). For example, the high porosity and interconnectivity are beneficial for establishing an optimal microenvironment that promotes cell-to-cell communication and facilitates seamless integration of the scaffold into the adjacent tissue at the implant site (Pina et al., 2015). Pore sizes can be categorized into nanoscale (nano-roughness, <100 nm), microscale (micro-roughness, 100 nm–100 μm), and macroscale (100 μm–millimeters) (Vagaská et al., 2010). Different pore sizes may influence distinct cellular processes: nanoscale pores have been shown to play a critical role in collagen fiber formation and ECM deposition, whereas macropores are essential for cell seeding, distribution, migration, and subsequent neovascularization *in vivo* (Smith et al., 2009). Different pore structures also influence the cellular biological behavior in response to biomaterials and the efficacy of tissue regeneration in periodontal applications (Table 9).

**TABLE 9:** Porosity in periodontal tissue regeneration.

Material	Reference	Pore size (μm)	Porosity (%)	Cell type	Method	Result
BG/GelMa	Mei et al. (2022)	300	-	hPDLSCs	A novel mesoporous (BG)/GelMA biomimetic scaffold by extrusion-based 3D printing	Enhances cell attachment and promotes osteogenic and cementogenic differentiation
PLGA	Luo J. et al. (2023)	30	-	BMSCs	Calcitonin gene-related peptide (CGRP)-loaded porous microspheres (PMs)	The porous architecture of PMs provides effective cell-carrying capacity and physical protection for BMSCs during transplantation
HA	Liu et al. (2016)	415 ± 20 203 ± 18	69 ± 0.5 50 ± 0.2	BMSCs	Porous collagen–hydroxyapatite scaffold and cross-linked collagen–hydroxyapatite scaffold	Results in significantly enhanced periodontal regeneration, characterized by the formation of new bone, periodontal ligament, and cementum in beagle dogs
CS	Liao et al. (2020)	20	-	hPDLCs	The mesoporous hydroxyapatite/chitosan (mHA/CS) composite scaffold	Inhibits the growth of periodontal pathogens and promotes the formation of bone and cementum-like tissue
TCP	Diloksumpan et al. (2020)	200	40.03 ± 1.78 51.14 ± 0.78	hPDLCs	The long-term *in vivo* performance of printed hydrogel–ceramic composites made of methacrylated-oligocaprolactone-poloxamer and low-temperature self-setting calcium phosphates is assessed in a large animal model	Stimulates bone formation

### 4.4 Drug delivery

Various forms of polymeric scaffolds for cell and drug delivery are available: (1) a conventional, three-dimensional porous matrix, (2) a nanofibrous matrix, (3) a thermosensitive sol–gel transition hydrogel, and (4) porous microspheres. Scaffolds provide a suitable substrate that supports cell attachment, proliferation, migration, and differentiated function. Scaffold matrices enable targeted drug delivery with high loading capacity and efficiency. Furthermore, incorporating therapeutic agents, such as anti-inflammatory inhibitors and antibiotics, into scaffolds can help prevent postoperative infection and manage related diseases over an extended period. Additionally, scaffolds can deliver biological signals—such as adhesion peptides and growth factors—to guide cells toward the desired differentiation state, maintain their viability, and support sustained growth (Garg et al., 2012). Given the tissue heterogeneity and antibacterial requirements inherent in the periodontal tissue regeneration process, the drug delivery capabilities of biomaterials play a critical role (Table 10).

**TABLE 10:** Drug delivery in periodontal tissue regeneration.

Materials	References	Drug	Method	Result
Hydrogels/lipid nanoparticles	Dos Santos et al. (2024)	Grape seed extract and simvastatin	Hierarchical nanostructured 3D-printed bilayer membranes that serve as dual-drug delivery nanoplatforms	Enhances alveolar bone regeneration and suppresses inflammation in a periodontal defect model
PCL/polyvinyl alcohol (PVA)	Limoee et al. (2019)	Ibuprofen	PCL and PVA were fabricated into electrospun fibers using an electrospinning process, with ibuprofen loaded separately into either the PCL or the PVA component	The controlled and sustained release of ibuprofen is achieved from both PCL and PVA/COL-loaded membranes, as an anti-inflammatory agent
Pluronic F127 (PF-127) and hyaluronic acid methacrylate (HAMA)	Liu Y. et al. (2024)	Spermidine-modified mesoporous polydopamine nanoparticles (M@S NPs)	A double-network hydrogel composed of PF-127 and HAMA) loaded with M@S NPs	PH/M@S effectively reduces the bacterial load in a rat model of periodontitis, alleviates local inflammation, and inhibits alveolar bone resorption
CS	Wang et al. (2025)	Melatonin	A composite of chitosan and mesoporous wollastonite loaded with melatonin	The scaffold demonstrates excellent biocompatibility in the chick embryo chorioallantoic membrane CAM model, with no signs of inflammation or necrosis, and exhibits pro-angiogenic properties, promoting extensive vascularization
Hydrogels	Zhang S. et al. (2023)	Spermidine	Developing a bi-crosslinking viscoelastic hydrogel (Alg-PBA/Spd) by integrating phenylboronic acid-modified alginate with an anti-inflammatory agent (spermidine) through borate ester and B–N coordination bonds	Promotes the deposition of periodontal collagen and accelerates the repair of periodontal damage
BG	Yao et al. (2022)	Simvastatin	Preparing an alginate-modified MBG	ALG significantly improves drug loading efficiency, prolongs drug release duration, and facilitates the orderly deposition of apatite on the surface of MBG

## 5 Discussion

With the in-depth application of biomimetic materials in tissue engineering, their use in periodontal tissue regeneration has become widespread. This article reviews the application of biomimetic materials and cells in periodontal tissue regeneration after reviewing the literature.

Biomimetic materials can be classified in various ways. Based on the extent to which the material or system mimics natural structures or functions, they can be categorized as bio-inspired, bio-derived, or biomimetic. Bio-inspired materials are not directly derived from biological sources but are synthetically designed to incorporate key functional elements found in nature (Semino, 2008). The inspiration often comes from specific chemical, physical, or topological cues, such as RGD-modified alginate. Bio-derived materials are obtained directly from biological sources such as animals, plants, or humans. They inherently possess many of the essential biological signals but often require processing to remove immunogenic components, such as dECM, collagen, fibrin, and hyaluronic acid. Biomimetic materials are designed not only to present biological cues but also to dynamically respond to their environment in a manner that mimics living systems. These materials often blur the distinction between a material and a machine, such as self-healing hydrogels. Biomimetic materials can be categorized into natural and synthetic polymer systems based on the origin of their polymer chains. Natural polymeric systems are derived from natural sources. They offer excellent biocompatibility and bioactivity; however, they may exhibit issues related to batch-to-batch variability, immunogenicity, and mechanical strength. Examples include collagen, fibrin, silk fibroin, HA, alginate, and chitosan. Synthetic polymeric systems are man-made polymers. They offer precise control over material properties, such as degradation rate, stiffness, and structure, as well as high reproducibility; however, they are often inert and lack biological recognition. Common examples include PEG, PLGA, PCL, and PLA. The selection of the appropriate system depends entirely on the specific application: a simple cell delivery vehicle may utilize a pure natural polymer, whereas a complex organoid model may require a sophisticated, stimuli-responsive synthetic hydrogel engineered with biomimetic intelligence.

Calcium phosphate biomimetic materials have garnered extensive attention in the field of dental implants because of their excellent bone conductivity and bone regeneration capabilities. These materials are chemically and structurally analogous to natural bone, making them ideal for repairing bone defects in periodontal tissue. Furthermore, CaP materials can be combined with other biomimetic materials by applying coatings to enhance the efficiency of tissue regeneration. Polymer materials exhibit biodegradability, biocompatibility, antibacterial properties, low antigenicity, moisture absorption, and moisture retention. These features make them ideal scaffolds for oral, craniofacial, and tissue-engineering applications. Polymer materials are widely utilized as synthetic biomimetic materials for drug and growth factor delivery systems and barriers for GTR and GBR. Hydrogels have advantages over many other biological materials owing to their superior physical and chemical properties. They possess high structural plasticity, enabling them to conform to the personalized architecture of the periodontal tissue. Additionally, hydrogels demonstrate excellent biocompatibility with various cell types and serve as effective sustained-release carriers for controlled drug delivery at different time points. Their printability can also be integrated with 3D printing technology in combination with other biomimetic materials, facilitating their application in periodontal tissue regeneration. dECM is extracted from natural tissues and exhibits excellent biocompatibility and cell-induction capabilities, providing an optimal microenvironment for the regeneration of host stem cells. The dECM hydrogel combines the high plasticity of hydrogel materials, enabling it to carry drugs with sustained-release properties, while effectively adapting to the personalized structural requirements of the periodontal tissue. Hard tissue-derived ECM demonstrates strong osteogenic/dental induction abilities, particularly odontogenic TDM, which not only possesses favorable physicochemical properties but also robust biological induction capabilities, making it a promising biological material for periodontal tissue regeneration.

The aforementioned biomimetic materials have played a significant role in periodontal tissue regeneration, yet they are associated with certain limitations. First and foremost, periodontal tissues—which comprise the gingiva, periodontal ligament, cementum, and alveolar bone—are essential for supporting and anchoring teeth within the jawbone. However, regenerating the complex structural architecture of these tissues remains a major challenge. To address this, scaffolds must be carefully designed and fabricated according to specific clinical requirements to fully realize their functional potential. Novel processing technologies aim to achieve structural features that mimic the extracellular matrix at multiple levels, strategies to emulate cell–extracellular matrix interactions, and biological delivery approaches to recapitulate signaling cascades or developmental and wound-healing programs (Ma, 2008). The rapid evolution of bioprinting has accelerated traditional regenerative medicine, making the fabrication of multilayered scaffolds—essential for targeting the periodontal ligament—conceivable. Physiological mechanical loading is fundamental for generating this complex anatomical structure *ex vivo* (Roato et al., 2022). The 3D bioprinting technology, particularly when employing biomaterials embedded with living cells and bioactive molecules, enables precise and controllable modulation of key structural parameters, including porosity, interconnectivity, pore size, and permeability. This additive manufacturing approach builds complex 3D architectures by sequentially depositing cell-laden hydrogels or other biomaterials in a layer-by-layer manner. Although various 3D bioprinting techniques have been developed, each has distinct advantages and limitations. Therefore, selecting an appropriate 3D bioprinting method that can accurately replicate the intricate structure and functional characteristics of native periodontal tissues is critical for reconstructing a conducive regenerative microenvironment and promoting effective tissue regeneration. Furthermore, achieving mechanical properties that closely match those of natural periodontal tissues remains a significant challenge. Mechanical loading induces proper alignment of the fibers that constitute the periodontal ligament and maintains tissue homeostasis, whereas excessive loading or inadequate adaptation to mechanical stimuli may partially contribute to aberrant tissue regeneration when using PDLSCs (Roato et al., 2022). With respect to mechanical performance, scaffolds should exhibit Young’s modulus and compressive strength comparable to those of native periodontal tissues. Specifically, physical properties of biomaterials—such as pore size, porosity, particle size, and mechanical strength—play a crucial role in regulating cell attachment and proliferation. For instance, high porosity and interconnectivity facilitate the establishment of an optimal microenvironment that enhances cell-to-cell communication and promotes seamless integration of the scaffold into surrounding tissue at the implant site.

## 6 Conclusions and future perspectives

Owing to the complex tissue structure and diverse cell composition of periodontal tissue, biomimetic materials used for periodontal tissue regeneration must possess excellent biological properties. They should not only mimic the ECM, participate in the release of signaling molecules, and regulate stem cell behavior but should also exhibit optimal physical and chemical characteristics, such as appropriate mechanical strength, suitable porosity, and a degradation rate compatible with tissue regeneration. Although the application of biomimetic materials in periodontal tissue regeneration has been extensively studied, several key challenges remain: (1) enhancing the biocompatibility of biomimetic materials to more effectively induce the differentiation of odontogenic stem cells; (2) optimizing the physical properties of biomimetic materials to better reconstruct the sandwich-like structure of natural periodontal tissue; (3) developing simpler and more efficient material preparation and preservation methods; (4) optimizing the mechanical properties of the scaffold, which is critically important for both tissue regeneration and the maintenance of long-term physiological function; (5) leveraging 3D printing technology to adapt to the personalized architecture of periodontal tissue by improving the printability of various biomimetic materials and enhancing printing accuracy—this represents a promising research direction; and (6) expanding the application of dECM, which lays a solid foundation for clinical research on periodontal tissue regeneration, although clinical data remain limited and further studies are needed. Although most studies have yielded encouraging results, extensive clinical trials are required to confirm their efficacy. In summary, although biomimetic materials have shown significant promise for periodontal tissue regeneration, substantial efforts are required to translate these findings into routine clinical practice.
